# Polishing Approaches at Atomic and Close-to-Atomic Scale

**DOI:** 10.3390/mi14020343

**Published:** 2023-01-29

**Authors:** Zhichao Geng, Ning Huang, Marco Castelli, Fengzhou Fang

**Affiliations:** 1 Centre of Micro/Nano Manufacturing Technology (MNMT-Dublin), University College Dublin, D04 V1W8 Dublin, Ireland; 2 State Key Laboratory of Precision Measuring Technology and Instruments, Laboratory of Micro/Nano Manufacturing Technology (MNMT), Tianjin University, Tianjin 300072, China

**Keywords:** polishing, roughness, sub-nanometer, atomic and close-to-atomic scale, ACSM

## Abstract

Roughness down to atomic and close-to-atomic scale is receiving an increasing attention in recent studies of manufacturing development, which can be realized by high-precision polishing processes. This review presents polishing approaches at atomic and close-to-atomic scale on planar and curved surfaces, including chemical mechanical polishing, plasma-assisted polishing, catalyst-referred etching, bonnet polishing, elastic emission machining, ion beam figuring, magnetorheological finishing, and fluid jet polishing. These polishing approaches are discussed in detail in terms of removal mechanisms, polishing systems, and industrial applications. The authors also offer perspectives for future studies to address existing and potential challenges and promote technological progress.

## 1. Introduction

Polishing is a process of creating a smooth and scratchless surface by using mechanical, chemical, and electrochemical approaches for reducing surface roughness and enhancing the workpiece’s strength [1,2]. Roughness directly determines the surface functional performance, and it is usually handled in the final process of machining, namely polishing.

**Figure 1:**
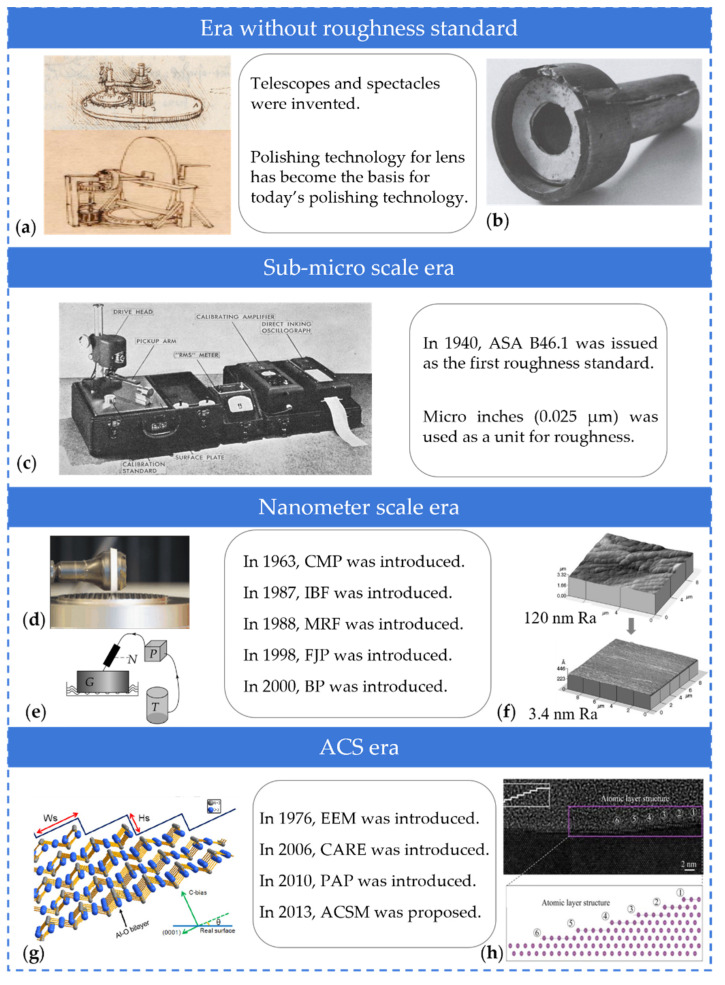
The historical development of roughness scale in polishing technology: (**a**) Leonardo da Vinci drew machines for manufacturing and polishing glass lenses. Reproduced with permission from [18]; (**b**) Galileo Galilei created a lens-polishing apparatus. Reproduced with permission from [19]; (**c**) A surface analyzer in 1950. Reproduced with permission from [8]; (**d**) Abrasive polishing tool for structured surfaces. Reproduced with permission from [20]; (**e**) Schematic diagram of the closed-loop set-up for FJP [12]; (**f**) Atomic force microscope (AFM) images of mechanically polished diamond films. Reproduced with permission from [10]; (**g**) Atomic models of hexagonal crystal step-terrace structure. Reproduced with permission from [21]; (**h**) Cross section transmission electron microscopy image of mechanochemical wear region and visualization of the atomic step edge [22].

The origins of polishing date to the Stone Age. Sandstones were utilized as polished stones as early as 4800~4600 BC [3]. Since then, polishing has evolved through four distinct eras (Figure 1), distinguished by the roughness scale that each polishing approach can achieve:

(1) Era without roughness standard: telescopes and spectacles were invented during the renaissance. Although there was no standard for roughness at that time, as early as 1634, it was already realized that polishing was not just cleaning the glass, but reducing the roughness, such as the cloth is shaved by the cropper [4]. The manufacture of lenses, prisms, and mirrors laid the groundwork for the development of polishing technology. In the 19th century, the “trial and error” production method is common to fabricate microscopes. Carl Zeiss and Ernst Abbe introduced diffraction limit, Abbe number and measured Abbe error to measurements, which led to a qualitative change in microscope polishing technology [5,6].
(2) Sub-micro scale era: after the second industrial revolution, optical theory and measuring technologies have propelled polishing into the era of standardization. The first roughness standard, ASA B46.1, was issued in 1940. Hereafter, roughness down to 9 µin in Ra (0.23 μm) [7] and 30 µin in RMS (0.76 μm) [8] were realized for rubberized seal and cast dental gold alloy, respectively. In this period, µin is a common unit for evaluating roughness and the highest precision of roughness in polishing was considered to be 0.5~5 µin [9]. It should be noted that Ra and Rz were not used as roughness parameters in the mid-twentieth century. Instead, they used *h*_ave_ and *h*_max_, where *h*_ave_ is similar to Ra and *h*_max_ is similar to Rz.
(3) Nanometre scale era: in the second half of the 20th century, the invention of precision polishing technologies including chemical mechanical polishing (CMP), ion beam figuring (IBF), magnetorheological finishing (MRF), fluid jet polishing (FJP), and bonnet polishing (BP) allowed the roughness achieved by polishing to reach the nanometre scale, achieving 3.4 nm in Ra for diamond [10], 2 nm in *R*_max_ for metal mirror surfaces made from Cu/Al alloys [11], and 1.6 nm in RMS for BK7 [12].
(4) Atomic and close-to-atomic scale (ACS) era: the maturation of computer numerical control (CNC) technology and the diversification of measurement techniques have led to a quantum jump in polishing technology at the sub-nanometer scale. Roughness values of 0.5 nm in RMS for tungsten [13], 0.5 nm in Ra for silicon nitride [14], and 0.15 nm in RMS for polysilicon [15] were realized. The sub-nanometer roughness indicates an upgrade from precision polishing to what we may define as “ultra-precision” polishing and entered the era of ACS [16,17].

Polishing at ACS is defined as a series of methods to realize surface roughness in Ångström level, where the material removal mechanisms are established by quantum mechanics. Using current polishing procedures, roughness in Ångström level represents the highest level of surface finish achievable.

**Figure 2:**
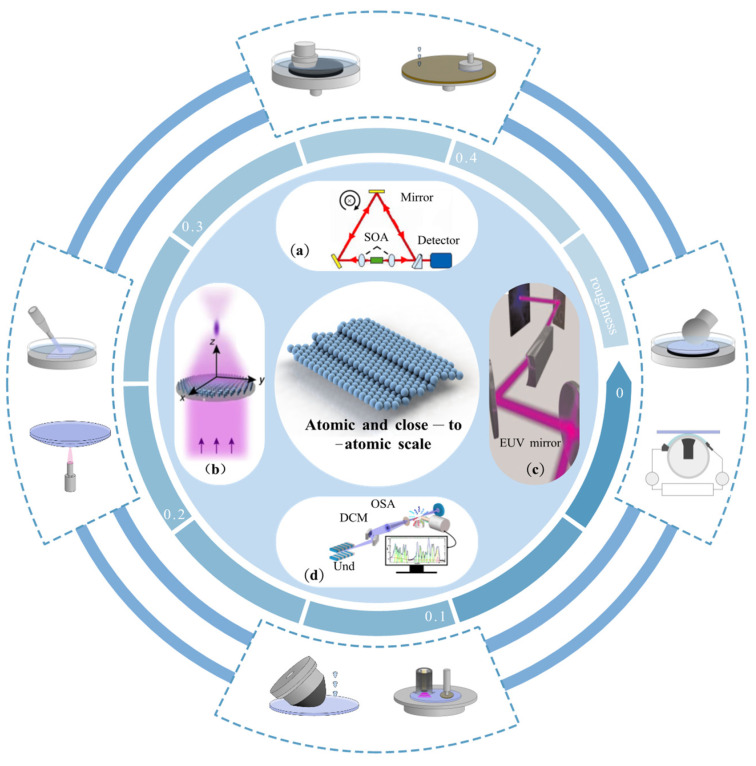
The demands and polishing approaches at ACS: (**a**) Measurement apparatus of the ring laser gyroscopes [40]; (**b**) Schematic drawing of focusing by a deep ultraviolet [41]; (**c**) Schematic of the tabletop extreme ultraviolet microscope [42]; (**d**) Schematic representation of an X-ray microprobe. Reproduced with permission from [43].

The chart of machining accuracy over time proposed by Taniguchi in 1983 [23] is now verified in practice [24]. Atomic and close-to-atomic scale manufacturing (ACSM) is the next generation of manufacturing technology and will be the leading trend in developing Manufacturing III [25]. Roughness in Ångström level is not only essential, but more importantly, achievable through polishing at ACS [26]. For instance, substrates mounted in ring laser gyroscopes are required to have a surface with a roughness of 0.5 nm in Ra [24]. For a hard X-ray system, 0.2 nm in RMS is required for mirrors [27,28]. In the field of ultraviolet lithography, 0.3 nm in RMS [29] and 0.15 nm in RMS [30,31] are needed by the lens. The roughness of a PC hard disc must be less than 0.05 nm in Ra to achieve a storage density larger than 500~1000 Gb/in^2^ [32], as shown in Figure 2.

The above examples indicate how the demands for polishing at ACS have become increasingly stringent [33], with the development of information technology [32], laser technology [34], optical industry [35,36], electronic power devices [37], and physical sciences [38,39].

To date, many reviews have been conducted on polishing techniques. Some of them focused on a particular material (silicon [44], sapphire [45], quartz [46], etc.) or machining methods (laser polishing [47,48], shear-thickening polishing [49], electrochemical mechanical polishing [50], etc.). As the state-of-the-art of processing technology, polishing at the ACS needs to be reviewed urgently. In this article, we will present the polishing approaches that have yielded sub-nanometer roughness in the past half century and explore the future development of ACS polishing.

**Figure 3:**
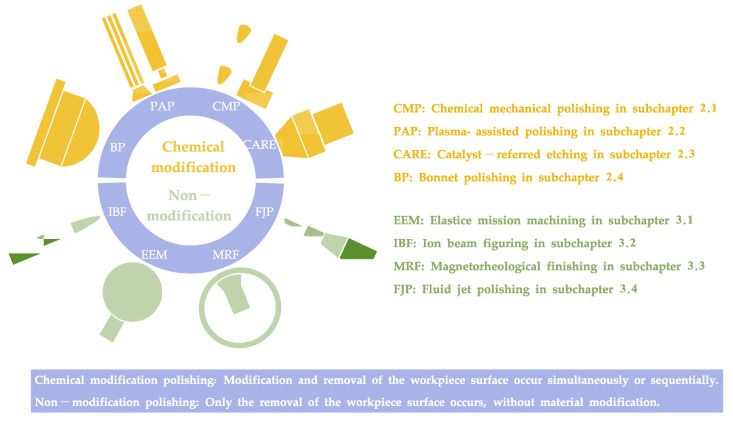
The classification of polishing approaches at ACS.

Polishing techniques were categorized as chemical modification and non-modification polishing approaches in this paper. Chemical modification polishing involves modifying and removing the surface material of the workpiece. Non-modification polishing refers to the removal of the workpiece material without material modification. Unlike other classification approaches (compliant and conventional polishing [51], dry and wet polishing [52], etc.), this classification is proposed to realize ACS efficiently on complex surfaces, namely freeform surfaces by serially combined processes of chemical modification polishing and non-modification polishing, as shown in Figure 3.

## 2. Chemical Modification Polishing Approaches

### 2.1. Chemical Mechanical Polishing

Robert introduced CMP to polish semiconductor materials in 1965 [53]. The International Business Machines Corporation (IBM) began utilizing CMP technology in dynamic random access memory manufacturing in 1988 [54]. Since then, IBM [55] and other researchers have investigated and progressed CMP technology continuously.

#### 2.1.1. Removal Mechanism

**Figure 4:**
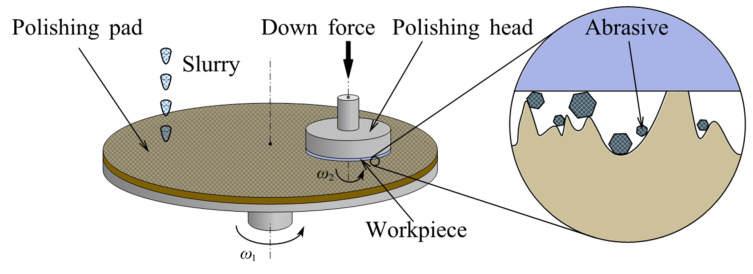
The schematic diagram of CMP [58].

CMP is a technique for atomic-level removal. The chemical reaction of the polishing slurry and the mechanical interaction of the abrasive are combined to remove material [56,57]. The workpiece is fixed on the polishing workhead spindle and loaded downward against the polishing pad. Both the polishing head and pad are driven by servo motors with adjustable speed. The chemical action of the polishing slurry softens a thin layer on the workpiece surface, which is subsequently removed by the mechanical interaction of upcoming abrasives to obtain an ultra-smooth surface [58], as shown in Figure 4.

The removal mechanism of CMP was studied from the following three aspects, including chemical reaction, mechanical interaction, and edge effect.

**Figure 5:**
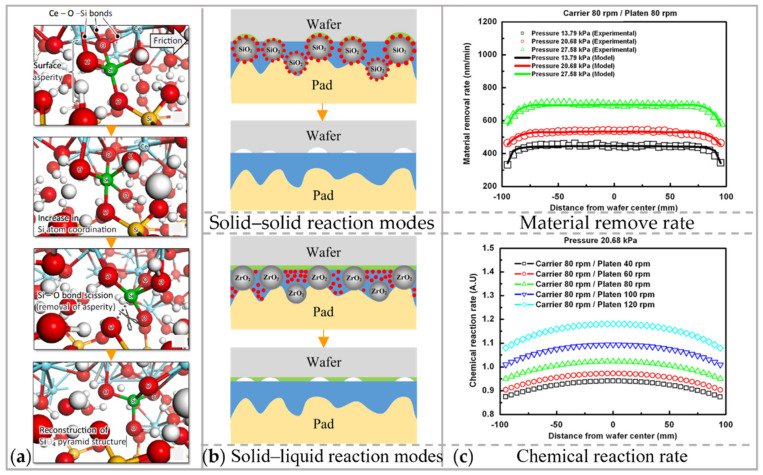
The removal mechanism in CMP from (**a**) Chemical reaction analyzed by first principles molecular dynamics. Reproduced with permission from [60]; (**b**) Chemical reaction divided into solid–solid reaction and solid–liquid reaction. Reproduced with permission from [61]; (**c**) Mechanical interaction. Reproduced with permission from [65].

(1) The chemical reaction modifies the surface material of the workpiece to facilitate subsequent mechanical removal. In the early 1990s, Cook [59] proposed the chemical bonding removal model. It describes how abrasive atoms/molecules interact with workpiece atoms/molecules to form chemical bonds. Based on the chemical bonding removal model, the chemical modification of Si was analyzed using molecular dynamics. For instance, when CeO_2_ was used as the abrasives, a Ce-O-Si bridging bond formed at the interface between workpiece and abrasive particle, as illustrated in Figure 5a. This condition causes instability of the pyramidal structure of the material, thus breaking the original Si-O bond [60]. This instability of the bridging bonds on the workpiece surface provides the basis for removal by subsequent mechanical interaction.

To obtain a sub-nanometer roughness, it is necessary to ensure the uniformity of the reaction layer. Zhang et al. [61] divided the chemical reaction modes in CMP into solid–solid reaction (reaction between the workpiece and solid particles) and solid–liquid reaction (reaction between the workpiece and slurry). Due to the detachment of the chemical reactant from the mechanical abrasives, the chemical and mechanical actions can realize a balance in solid–liquid reaction mode. The depth of the soft reaction layer in the solid–liquid reaction is more uniform than that in the solid–solid reaction, resulting in a smooth surface, as shown in Figure 5b.

(2) Mechanical interaction removes the material directly after chemical modification. The Preston equation was employed to calculate material removal rate *MRR* under mechanical interaction [62]:

(1) MRR=KPV

where *P* and *V* denote the average contact pressure and the relative velocity between the workpiece and polishing pad, and *K* is the Preston coefficient to describe the effects of the chemical process, pad material, and abrasive type and size.

Equation (1) implies that the *MRR* is proportional to the contact pressure and relative velocity between the polishing pad and workpiece. The Preston equation is limited to solving the material removal rate distribution that is necessary to predict the form accuracy of the workpiece surface. The material removal rate distribution *MRR*(*x*, *y*) is given as [63,64]:

(2) $$MRR\left(x,y\right)=k\left(x,y\right)c\left(x,y\right)p_{av}v\left(x,y\right)$$

where *c*(*x*, *y*) is the locally relevant expression of the contact pressure distribution function. *p*_av_ is the average contact pressure, i.e., force divided by contact area.

To determine the average material removal rate of the materials that can be etched in the polishing slurry, it is necessary to consider chemical reaction rate distribution, as shown in Figure 5c. Equation (1) was modified by integrating the etching reaction between Cu and the polishing slurry [65]:

(3) $$\mathrm{MR}R_{\mathrm{avg}}=KP^{a}V^{b}{}_{\mathrm{re}.\mathrm{avg}}+e_{d.\mathrm{avg}}$$

where *e_d.avg_* is the average dynamic etch rate.

(3) The fluid floating and tool roll-off effect influences *P* in Preston equation. Although the erosion effect of the polishing fluid on the polished material is almost negligible [66], based on the Reynolds equation [67], fluid pressure affects the hydrodynamic lubrication properties of the slurry and the dynamic balance in CMP [68]. During the polishing process, the fluid floating effect caused by the hydrodynamics lifts the workpiece leading edge up. As a result, there is negative pressure at the edge of the workpiece [68]. Tool roll-off effect refers to the fact that as polishing pad progressively hangs over the edge of the workpiece, the pressure naturally diminishes due to the smaller and smaller contact area. This edge effect deteriorates the form accuracy of the workpiece.

According to the removal mechanism in CMP, both mechanical and chemical action play an equally crucial role in achieving material removal. Maintaining the balance between these two actions is the key to obtaining sub-nanometer roughness.

#### 2.1.2. Chemical Reaction Factors

**Figure 6:**
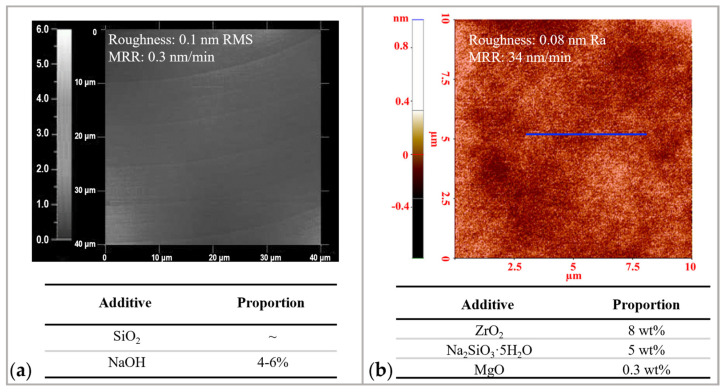
The CMP recipe and AFM image of polished YAG: (**a**) Commercial recipe with SiO2. Reproduced with permission from [73]; (**b**) Novel recipe with Na2SiO3·5H2O reagent. Reproduced with permission from [71].

The recipe of the CMP slurry determines the process and rate of chemical reactions. Typically, the recipe includes abrasive particles, oxidizers, surfactants, and deionized (DI) water [69]. Material removal is mostly determined by the type of abrasive particles. For example, SiO_2_ (colloidal silica) is conventionally used to polish yttrium aluminum garnet (YAG) with a relatively low material removal rate (0.3 nm/min [70]). Instead, Zhang et al. [71] used ZrO_2_ with Na_2_SiO_3_·5H_2_O and MgO as slurry to increase the material removal rate to 34 nm/min with ACS precision, as shown in Figure 6. The increase in material removal rate is due to the unbinding of chemical and mechanical action. In commercial polishing slurry, Si-OH is distributed on the surface of SiO_2_ particles [72], so that chemical reactions and mechanical actions are bonded to the same particles, resulting in lower efficiency. In the novel polishing slurry, Na_2_SiO_3_·5H_2_O replaces Si-OH to exercise the chemical action and ZrO_2_ replaces SiO_2_ to exercise the mechanical action. The two are carried out separately, which helps to achieve a balance between chemical and mechanical action.

The chemical reactions between the polishing slurry and the workpiece are determined by the surface charges. As a result, the material removal rate and roughness are determined by the acidity or alkalinity of the slurry [74]. For instance, the removal of Si was shown to be highest under alkaline conditions, as the OH termination increases with solution pH and strong polarization weakens Si–Si back bonds [75]. Acid slurries, instead, have been shown to reduce roughness while polishing tungsten alloy. For example, citric acid-based slurry balances mechanical and chemical processes and prevents grain boundary steps from developing, thus offering a superior surface finish [76]. Li et al. [74] used acid colloidal silica to obtain sub-nanometer roughness for YAG crystal, showing that the acidic slurry facilitates chemical reactions and the elimination of surface scratches or damages. From this exploration, one can conclude that to obtain sub-nanometer roughness, the optimal pH of polishing slurry needs to be explored depending on the workpiece material.

#### 2.1.3. Mechanical Interaction Factors

**Figure 7:**
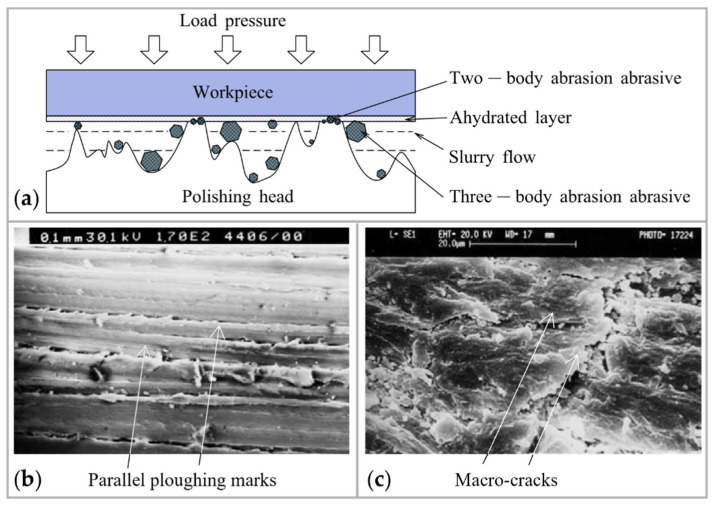
The contact model of the workpiece and polishing pad: (**a**) The schematic of CMP in micro view; (**b**) Scanning electron microscope (SEM) images of polyetherketone, under two-body abrasive abrasion, and (**c**) Three-body abrasive abrasion. Reproduced with permission from [77].

Mechanical interaction ultimately removes the material, also influencing the surface roughness. The mechanical abrasive process in CMP is divided into two-body abrasion and three-body abrasion. Under two-body abrasion, particles are trapped in the polishing pad, while the hard protuberances slide on the workpiece surfaces. Under the three-body abrasion model, abrasive particles are free to roll and slide [77], as shown in Figure 7a. Experiment [78] and simulation [79] showed that the material removal rate in three-body abrasion is one order of magnitude less than that for two-body abrasion. The micro-graphs, as shown in Figure 7b,c, indicated that there are parallel ploughing marks on the abraded surface under two-body abrasion, while the matrix damage is removed due to the formation of micro-cracks under three-body abrasion [77]. The transition from two-body to three-body abrasion mode is determined by polishing pad pressure. Lower contact pressures in CMP will reduce two-body abrasion and increase three-body abrasion. Sub-nanometer roughness is more easily achieved due to the smaller indentation depth of the abrasive particle under three-body abrasion.

**Figure 8:**
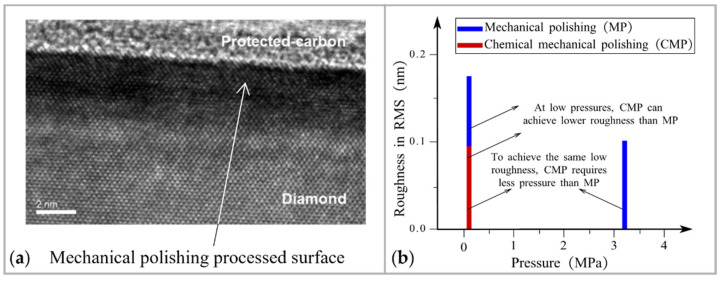
The effect of pressure on SCD roughness: (**a**) Cross-sectional transmission electron microscopy image of the polished diamond surface. Reproduced with permission from [83]; (**b**) The roughness achieved in mechanical and CMP at different pressure.

Pressure has a direct impact on roughness when polishing single-crystal diamond (SCD). If there is only mechanical interaction without chemical reaction to modify the top of the workpiece surface, this polishing is called mechanical polishing. Unlike mechanical polishing, which requires considerable pressure to achieve material removal [80], CMP can polish high-hardness materials with ACS precision at atmospheric pressure. In mechanical polishing with pressure as high as 3.27 MPa, AFM image shows that the roughness of SCD can be 0.105 nm in RMS [81,82,83]. When the pressure lowered to 0.10 MPa, scanning white light interferometric images shows that the roughness deteriorated to 0.172 nm in RMS [81,82,83], as shown in Figure 8a. However, the AFM image shows that CMP can achieve roughness as low as 0.09 nm in RMS at ambient pressure [84]. Due to the ACS precision obtained under ambient pressure, CMP simplifies the process of polishing SCD, as shown in Figure 8b.

#### 2.1.4. Characteristics

By combining chemical and mechanical actions, CMP can produce ultra-smooth surfaces with greater precision than machining equipment, as shown in Table 1.

It is noted that CMP has encountered difficulties such as:

(1) While softening the workpiece surface, the polishing slurry also has a corrosive effect on the polishing pads, resulting in more frequent replacement of polishing pads and higher costs.
(2) During the polishing of metals, the workpiece is easily scratched by the abrasive grains due to the low hardness, making it difficult to achieve sub-nanometer roughness [91,92,93]. Trial and error are inevitable to find an appropriate slurry recipe when CMP is applied to new metal material.
(3) Low contact pressure is necessary to achieve low roughness, but unavoidably affects removal efficiency. As loose abrasive particles rarely slide on the workpiece surface under low contact pressure, they spend about 90% of their time rolling according to the three-body abrasion mode [77].
(4) The workpiece roughness under three-body abrasion is sensitive to the abrasive size. When the abrasive size is not uniform, the mechanical material removal is carried out by a small number of large abrasives, probably resulting in the formation of scratches, pits, and other damage.

### 2.2. Plasma-Assisted Polishing

Yamamura et al. [94] proposed plasma-assisted polishing (PAP) for the finishing of difficult-to-machine materials in 2010. PAP with atmospheric pressure plasma and soft abrasives is widely considered to be an ultra-precision polishing approach [95].

#### 2.2.1. Removal Mechanism

**Figure 9:**
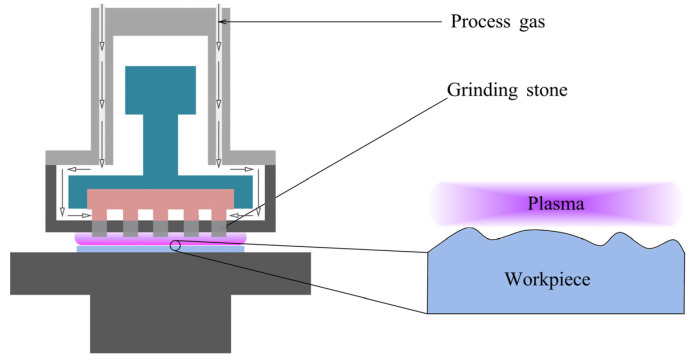
The schematic diagram of PAP [95].

Figure 9 shows the schematic of the PAP setup. Workpiece surface modification by plasma irradiation alternate with material removal by soft abrasive in the PAP process [95].

**Figure 10:**
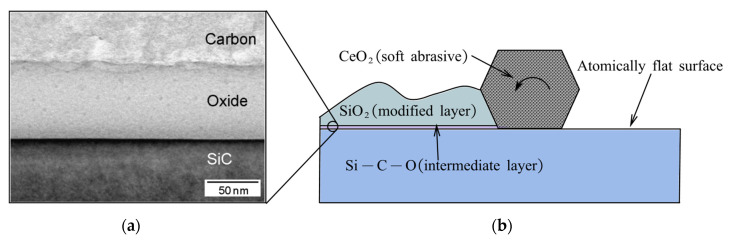
The removal mechanism in plasma-assisted polishing: (**a**) Cross sectional transmission electron microscopy images of H_2_O containing plasma irradiated 4H-SiC surface. Reproduced with permission from [97]; (**b**) The removal process in PAP at microscopic.

Modification of the surface status is carried out by means of plasmas with various types of additives [96]. After modification, the upper layers of the modified surface are instantly removed by mechanical interaction, as shown in Figure 10. In the process of mechanical removal, fixed type grinding stone mounted on the tip of a spindle is preferred to achieve a damage-free surface, otherwise, the agglomeration phenomenon introduced by loose-held-type abrasive will produce scratches and pits [95].

**Figure 11:**
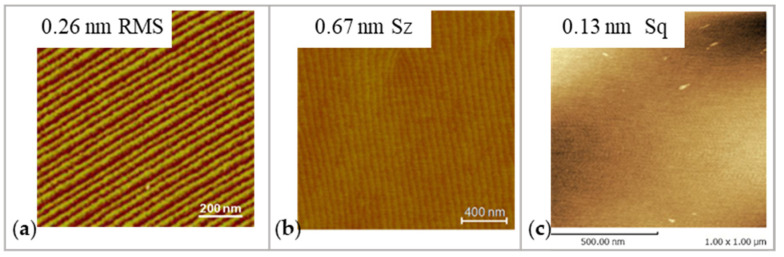
The AFM images of PAP-processed workpiece: (**a**) SiC. Reproduced with permission from [97]; (**b**) GaN. Reproduced with permission from [99]; (**c**) SCD. Reproduced with permission from [100].

The specific processing mechanism of PAP depends on the individual material. For SiC, the surface is oxidized under plasma irradiation to a soft modification layer composed of SiO_2_ and Si-C-O [98]. The interface between the modified layers and bulk material is atomically flat. A sub-nanometer roughness is achieved on the SiC surface when the chemically modified layer is removed mechanically, as shown in Figure 11a.

For GaN, the surface is fluorinated into the GaF_3_ soft modified layer by CF_4_ plasma irradiation. There is an atomically flat interface between the modified layer and bulk material because the dislocation sites on the surface are preferentially modified. An atomic-scale precision surface without scratches is generated when CeO_2_ (softer than GaN) removes the modified layer [99], as shown in Figure 11b.

For SCD, argon-based plasma containing water vapor is utilized in PAP to modify not only the SCD surface but also the grinding stone surface. Here, it is reported that a dehydration condensation reaction was formed between the modified surfaces of the workpiece and the grinding stone terminated by OH, leading to the removal of SCD carbon. This removal does not cause any graphitization or amorphization, ensuring that the SCD surface acquires a sub-nanometer roughness [100], as shown in Figure 11c.

The mechanism of the surface modification presents several variations in the PAP process. It can occur via oxidation, fluorination, or OH termination for SiC, GaN, and SCD, respectively. Therefore, the specific processing parameters vary, as shown in Table 2.

#### 2.2.2. Processing Parameter in PAP

**Figure 12:**
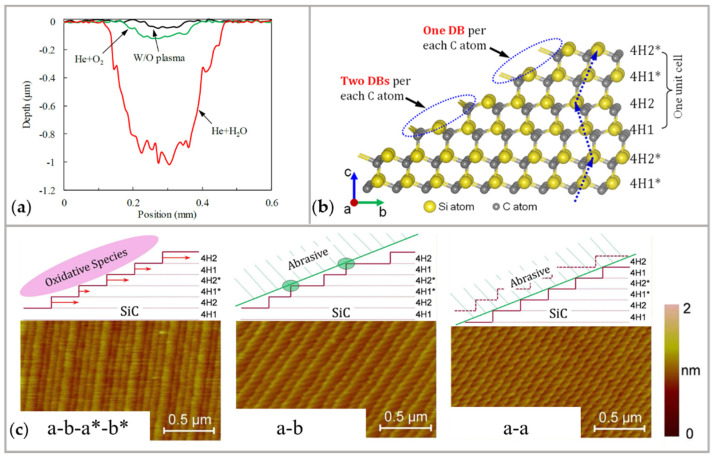
The influence of parameters on polishing results in PAP: (**a**) Comparison of removal depth when using O_2_ and H_2_O as precursor gas after ball-on-disc test [94]; (**b**) The crystal structure of 4H-SiC [101]; (**c**) Generation mechanism and AFM images of surfaces step–terrace structure of polished SiC (a-b-a*-b* type: step-terrace structure with a narrow terrace, a wide terrace and two terraces of intermediate width; a-b type: step-terrace structure with alternating narrow and wide terrace pairs; a-a type: step-terrace structure with a uniform terrace width) [101].

Due to the varying chemical modification capabilities of the radicals created by the process gas, the kind of precursor gas determines the efficiency of the modification. For instance, ball-on-disc tests showed that the SiC material removal efficiency was higher when H_2_O was used as the precursor gas than when using O_2_, as shown in Figure 12a. This is because the OH radicals generated from H_2_O have a higher oxidation capacity than the O radicals generated from O_2_ [95]. Specifically, the oxidation potential of OH is 2.80 V and that of O is 2.42 V.

The grinding stone performs a mechanical action following the chemical modification. The rotation speed of the grinding stone produces different step–terrace structures on the workpiece [95]. Here, changes in rotation speed result in a different proportion of chemical modification and mechanical removal for a given modification rate. 4H-SiC contains four types of oxidized terraces (4H1, 4H2, 4H1*, and 4H2*) with varying widths, as shown in Figure 12b. This leads to the formation of three types of steps: (1) In the case when chemical modification plays the main role, only the modified layer is removed. After removing the modified layers, the a-b-a*-b* type step–terrace structure is generated. (2) When the mechanical removal is equivalent to the chemical modification, wide terraces are preferentially removed, thus, the step–terrace structure is changed to the a-b type. (3) When mechanical removal is the primary factor, all the terraces are in uniform contact with the abrasive particles after the oxide layer is removed. Therefore, the uniform a-a type step–terrace structure is generated, as shown in Figure 12c.

#### 2.2.3. Combination with Other Polishing Approaches

To extend the plasma polishing application to materials with poor machinability such as CVD-SiC and GaN, the PAP and plasma treatment were combined with other polishing approaches.

**Figure 13:**
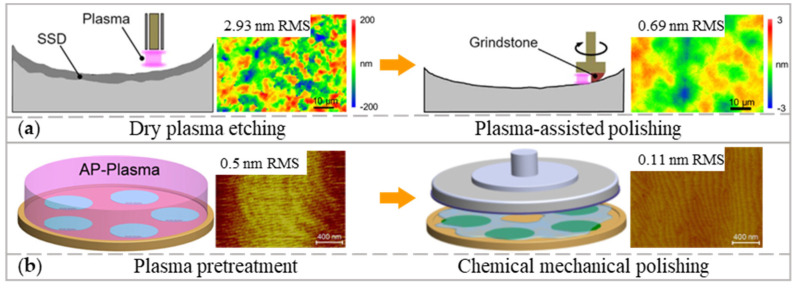
The combined process of the plasma treatment with (**a**) dry plasma etching. Reproduced with permission from [101]; (**b**) CMP. Reproduced with permission from [102].

A dry plasma etching process can efficiently remove the sub-surface damage and scratches formed by previous mechanical processes, but the surface roughness after dry plasma etching typically increases. PAP using resin-bonded grinding stones can decrease the roughness from 2.93 to 0.69 nm in RMS without causing sub-surface damage and scratches. The combination of the dry plasma etching process and PAP can realize the damage-free finishing of CVD-SiC substrates, as shown in Figure 13a [102].

Plasma pretreatment was also used to modify the surface of GaN [103]. The modified layer inhibits the formation of etch pits on the workpiece, which is a tricky issue in polishing GaN and the cause of high roughness. As depicted in Figure 13b, time-controlled CMP followed by plasma pretreatment could provide damage-free finishing and roughness of 0.11 nm in RMS.

#### 2.2.4. Characteristics

Mechanical polishing and plasma etching (e.g., Plasma Chemical Vaporization Machining, PCVM) are relatively efficient processes [104,105], but present a series of disadvantages when used individually, such as residual defects or high roughness [106,107]. PAP technique, instead, that combines the superior properties of mechanical polishing and PCVM [108] can achieve higher material removal rates, without sub-surface damage reaching sub-nanometer roughness, as shown in Table 3.

However, in PAP processing, there is still room for further research into the modification mechanism and process optimization.

(1) The atomically flat interface between the modified layer and bulk material is the key to obtaining ACS precision. Although an atomically flat interface has been observed by cross sectional transmission electron microscopy [95], there is a lack of specific research as to why this interface forms, as well as about the types or interfaces that can be formed on various materials. This severely limits the application of PAP. For example, the achievable roughness is only 3 nm in Sa when applied to AIN [110].
(2) The grinding stone surface in the PAP setup is planar, which limits the polishing of sloped surfaces.
(3) In the modification process of PAP, the precursor gas is one of the necessary factors. However, the stability of the gas flow is difficult to control in practice. The difficulty in controlling the chemical concentration limits the stability of the polishing.

### 2.3. Catalyst-Referred Etching

Catalyst-referred etching (CARE), an abrasive-free polishing approach [111], was investigated by Hara et al. in 2006 [112].

#### 2.3.1. Removal Mechanism

**Figure 14:**
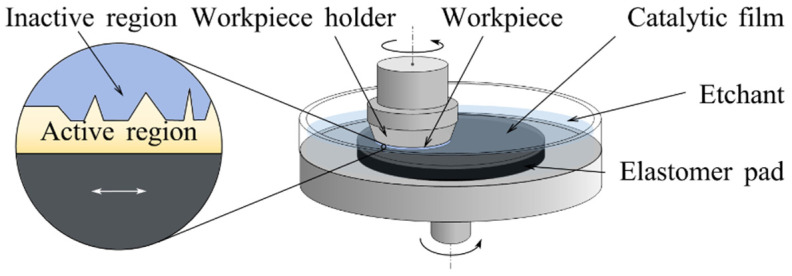
The schematic diagram of a CARE [114].

Figure 14 shows the setup of CARE. The workpiece and catalytic film that are fully immersed in the etchant independently rotate on each axis. The workpiece surface is in contact with the catalytic film surface. As the catalyst makes etching easier, the top part of the workpiece is in the active region and preferentially removed. In addition to the catalytic effect, the catalytic film surface also acts as a reference pad that statistically imprints its form accuracy to the planar workpiece surface [113].

CARE can be employed to improve the finishing of 4H-SiC surfaces down to the atomic scale. The flat and well-ordered workpiece surface processed by CARE was observed by transmission electron microscopy (TEM) [115]. High-resolution transmission electron microscopy (HRTEM) images indicated that there are alternating wide and narrow atomic-level terraces on CARE-processed surface [116].

The removal mechanism of CARE is mainly a chemical reaction. Low energy electron diffraction [117] images proved that there is no crystallographic damage caused by mechanical interaction on the CARE-processed surface. Arima presented the chemical reaction equation in CARE [118]. When the SiC is immersed in HF, the Pt surface was assumed to be flat, and only protrusions on the SiC surface are in contact with the Pt catalyst. The protrusions on SiC are removed at the anode by the following chemical reaction:

Cathode reaction (Pt):

(4) $$8H^{+}\to 4H_{2}+8h^{+}$$

Anode reaction (SiC):

(5) $$\mathrm{SiC}+4H_{2}O+8h^{+}\to {\mathrm{SiO}}_{2}+8H^{+}+{\mathrm{CO}}_{2}$$

(6) $${\mathrm{SiO}}_{2}+6\mathrm{HF}\to H_{2}{\mathrm{SiF}}_{6}+2H_{2}O$$

The kinematic characteristics are crucial to achieving sub-nanometer roughness. The workpiece is rotating around the axis at a speed different from the catalytic film. The workpiece is practically pushed against a flat surface, whereby only the protrusions on the workpiece surface are in contact with the flat catalytic film and are removed by chemical reaction to form a well-ordered surface.

#### 2.3.2. Expanding the Application

**Figure 15:**
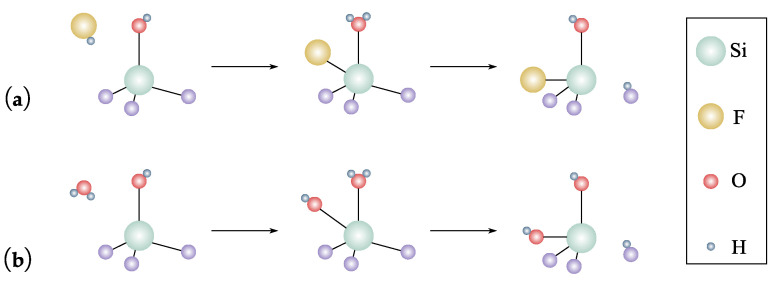
The schematic of reaction pathways of CARE with (**a**) HF; (**b**) pure water [113].

To introduce CARE to the electronics industry, Okamoto et al. [119,120] explored how to planarize 8° off-axis 4H-SiC substrates and the role of the smoother catalyst plate. To improve the material removal rate, the effect of processing pressure, rotating velocity [114], and the role of HF [121] were investigated. Specifically, the material removal rate increases as the velocity and pressure increase. Because of the low activation barrier for HF molecules adsorption, appropriately increasing the concentration of HF will improve the material removal rate as well. To reduce environmental pollution, pure water was tested as an alternative etchant to HF material removal was successfully achieved [122,123,124]. Similar to F^−^ in HF [113], OH^−^ in pure water is considered to induce indirect dissociative adsorption for achieving material removal of SiC, as shown in Figure 15.

To extend the process to materials other than SiC, studying the processing principles and applications of metal-assisted chemical etching (MACE) is recommended. This is due to CARE being originally inspired by the phenomenon that the etching of semiconductors in HF-based solutions is enhanced by noble metals [118].

**Figure 16:**
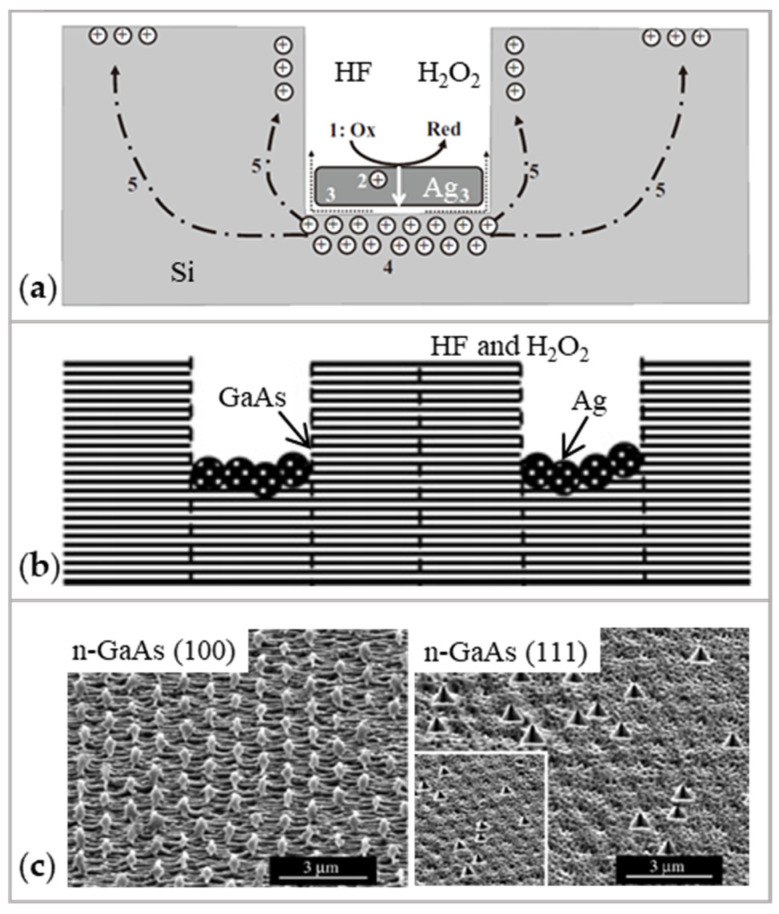
The MACE process: (**a**) The schematic diagram of processing Si [125]; (**b**) The schematic diagram of processing GaAs. Reproduced with permission from [129]; (**c**) The SEM micrographs of Ag-assisted etching for n-GaAs (100) and n-GaAs (111). Reproduced with permission from [129].

Si is a material commonly processed in MACE. During etching, H_2_O_2_, HF, and Ag act as the oxidant, etchant, and catalytic noble metal, respectively. Figure 16a shows the MACE processes. H_2_O_2_ is preferentially reduced at the surface of the Ag due to the catalytic activity, as shown in Equations (7) and (8). The Si is oxidized at the injection holes and dissolved at the Si/Ag interface by HF, as shown in Equations (9) and (10) [125].

Cathode reaction (Ag):

(7) $$H_{2}O_{2}+2H^{+}+2e^{-}\to 2H_{2}O$$

(8) $$2H^{+}+2e^{-}\to H_{2}↑$$

Anode reaction (Si):

(9) $$\mathrm{Si}+2H_{2}O\to {\mathrm{SiO}}_{2}+4H^{+}+4e^{-}$$

(10) $${\mathrm{SiO}}_{2}+6\mathrm{HF}\to H_{2}{\mathrm{SiF}}_{6}+2H_{2}O$$

GaAs is another material that can be processed with MACE. The recipes of catalyst, etchant, and oxidant are the same as when etching Si, as shown in Figure 16b. The parameters of etching Si and GaAs using MACE are shown in Table 4.

**Table 4:** The processed material with different catalysts, etchants, and oxidants using MACE.

Material.	Catalyst (Nobel Metal)	Etchant and Oxidant
Si	Au, Pt	HF and H_2_O_2_ [126]
Si	Pt	HF and H_2_O_2_ [127]
Si	Cu	HF and H_3_PO_3_ [128]
GaAs	Ag	HF and H_2_O_2_ [129]

Due to the similarity of material removal mechanism to MACE, it is possible to consider CARE to polish Si and GaAs by using noble metals Pt, Au, Cu, and Ag, and adding oxidants such as H_2_O_2_ and H_3_PO_3_.

#### 2.3.3. Characteristics

Table 5 mainly focused on how to use CARE to polish different types of SiC, the workpiece has realized roughness 0.1 nm or less in Ra or RMS. As shown in these studies, not only the microcracks were reduced [130], but also atomically flat [118] and well-ordered crystalline structures [115] can form on CARE-processed workpiece surfaces.

CARE still has the potential to be explored:

(1) Currently, noble metals (catalyst) and HF (etchant) are fundamental in CARE. The use of noble metals increases the cost of CARE. The use of environment-hazardous HF makes CARE difficult to be applied in industry [122]. Although some researchers applied pure water instead of HF, the material removal rate is 2 nm/h [122], which is very low compared with material removal rate 500 nm/h using HF [114].
(2) The ability to achieve sub-nanometer roughness in CARE relies on the accuracy of the catalyst film surface. While polishing off-axis SiC, the researchers have to lap the catalyst film by hand to minimize the degree of convexity [119]. Human intervention leaves uncertainty about the accuracy of CARE.
(3) Two difficulties arise if CARE is applied to curved surfaces: (a) The material exhibits anisotropy during etching [129], thus decreasing the precision, as shown in Figure 16c. (b) The direction of material removal depends on the crystal orientation of the workpiece [131], it is difficult to form a Gaussian distribution which could be used for surface figure error correction.

### 2.4. Bonnet Polishing

BP was introduced by Walker in 2000 [132]. BP technology is widely used as an ultra-precision polishing technique for difficult-to-machine materials to obtain sub-nanometer roughness [133,134].

#### 2.4.1. Removal Mechanism

**Figure 17:**
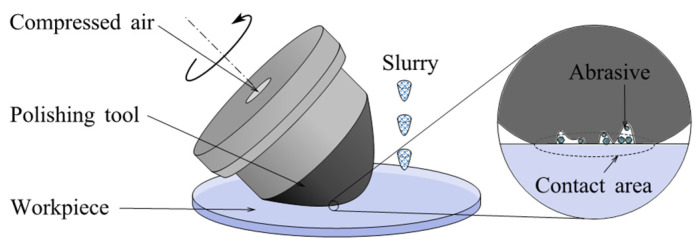
The schematic diagram of BP [135].

Figure 17 shows the operational schematic of BP. Polyurethane and other polishing cloths are chosen as a polishing pad to cover the spinning, bulged, and compliant polishing tool. This tool is shaped like a bonnet. During polishing, the tool rotates while a jet of abrasive slurry is directed at the interface to the workpiece. Workpiece and tool are moved relative to each other by multi-axis precision CNC systems resulting in complete freeform polishing. The offset of the workpiece to the tool and the internal pressure of the tool can be modulated independently to adjust the polishing pressure and contact area [130].

Preston equation is the foundation for solving the BP removal function [136]. Based on the Preston equation, Pan et al. [137] carried out a series of experiments and proposed the remove function:

(11) RR=ηkPV

where *η* is the interfacial friction coefficient, *k* is the Preston coefficient, and *P* and *V* are the pressure and relative velocity distribution on the contacting area, respectively.

**Figure 18:**
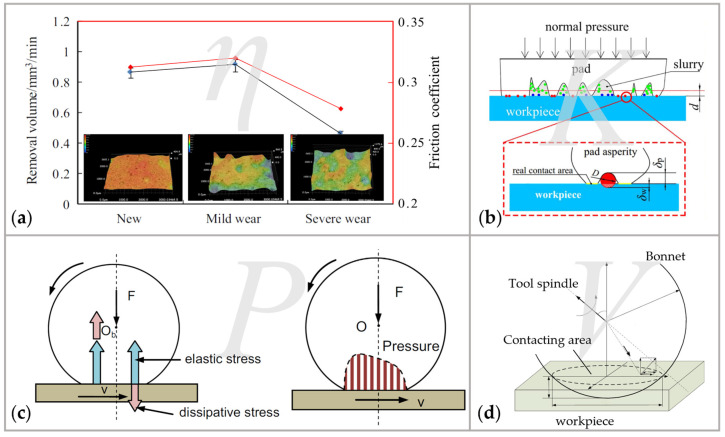
The material removal mechanism for BP: (**a**) The comparison between the removal volume and friction coefficient, and surface texture of bonnet tools with the three wear conditions. Reproduced with permission from [138]; (**b**) The contact behavior of a single abrasive particle with the workpiece and pad asperity [139]; (**c**) The sketch of pressure distribution for the rolling bonnet. Reproduced with permission from [133]; (**d**) The kinematic model of BP. Reproduced with permission from [137].

This model provides the following insights into the BP process:

(1) The variation trend of the friction coefficient *η* between the workpiece and bonnet tool is similar to that of the removal volume [138], as shown in Figure 18a. This positive correlation indicates that friction directly affects the material removal process. The frictional force between the workpiece and the bonnet tool is decreased during the polishing process, so that wear converts the pores on the tool surface to the glazing zone, which increases polishing quality.

(2) The Preston coefficient *k* is affected by the material of the workpiece, the concentration of the polishing slurry, and the number of abrasive particles [138]. The number of abrasive particles directly participating in the process is a key parameter, as one of the main causes of material removal is abrasive wear of the abrasive particles in the slurry [139], as shown in Figure 18b. Inside and outside the contact region, the abrasive particles are assumed to be distributed uniformly [140]. In this way, the active number of abrasive particles can be calculated [139]:

(12) $$N=A_{\mathrm{real}}\cdot {\left(\frac{\chi }{\xi }\right)}^{2/3}$$

where *A*_real_ is the real contact area, *χ* is the volume concentration of particles in the slurry, and *ξ* is the average volume of an individual particle.

(3) Pressure *P* in the contact area influences the surface deformations of the bonnet while the elastic sphere slides over a surface with the abrasive slurry, as shown in Figure 18c. The total pressure *σ_ij_* is a sum of the elastic part of the pressure and the dissipative $\sigma_{ij}^{el}$ part of the pressure $\sigma_{ij}^{dis}$ [133]:

(13) $$\sigma_{ij}^{el}=E_{1}\left(\varepsilon ij-\frac{1}{3}\delta_{ij}\varepsilon_{kk}\right)+E_{2}\varepsilon_{kk}\delta_{ij}$$

(14) $$\sigma_{ij}^{dis}=\eta_{1}\left({\dot{\varepsilon }}_{ij}-\frac{1}{3}\delta_{ij}{\dot{\varepsilon }}_{kk}\right)+\eta_{2}{\dot{\varepsilon }}_{kk}\delta_{ij}$$

where *ε_ij_* and are the strain ${\dot{\varepsilon }}_{\mathrm{ij}}$ and strain rates, respectively, *E*_1_ and *E*_2_ are the elastic material constants, *η*_1_ and *η*_2_ are the coefficients of viscosity, and *δ_ij_* is the Kronecker symbol.

(4) Only the velocity *V* on the contacting area influences the removal of the workpiece, as shown in Figure 18d. The relative velocity in the contact area is calculated as follows [137]:

(15) $$V=\sqrt{{\left(\frac{\left(R_{b}-h\right)\sin\rho n2\pi }{60}-\frac{y\cos\rho n2\pi }{60}\right)}^{2}+{\left(\frac{x\cos\rho n2\pi }{60}\right)}^{2}}$$

where *x* and *y* denote a random point on the contacting area, *R_b_* is the bonnet radius, *h* is the bonnet compression, *n* is the rotational speed of the bonnet tool, and *ρ* is the precession angle.

#### 2.4.2. Polishing System Development

OSL and Zeeko invented the first fully productionized machine, IRP200, to polish BK7 and achieve the roughness 0.5 nm in Ra [141]. The IRP 200 is equipped with a seven-axis CNC system [142], in which four axes control the workpiece motion and the other three axes control the polishing head. This multi-axis structure allows the BP approach to be applied to freeform polishing. Following their research on tool path planning [143], physical mechanisms [144], and control of edge mis-figure [145], Zeeko extended the range of IRP series: IRP 50 and IRP 100 [146] for small parts, IRP 400 and IRP 600 [147] for medium-sized parts, and IRP 1200 [148], IRP 1600, and IRP 2400 [149] for larger parts were developed.

#### 2.4.3. Tool Path Planning Approach

Although tribochemical modification occurs for both BP and CMP, it is more difficult for BP to achieve sub-nanometer roughness than CMP. In CMP, the workpiece and the polishing pad rotate around different axes. This special kinematic promotes low roughness. In the BP process, the roughness of the workpiece is highly dependent on the tool path planning.

**Figure 19:**
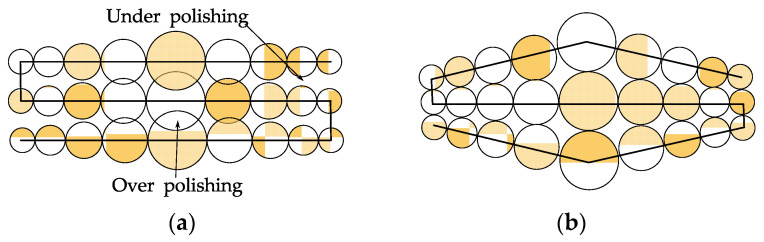
The schematic diagram of the polishing path: (**a**) Physically and (**b**) geometrically uniform coverage. Reproduced with permission from [150].

To avoid over-polishing and under-polishing in geometrically uniform coverage of the polishing path, Han et al. [150] proposed an efficient iterative approximation algorithm to create physically uniform coverage of the polishing path, as shown in Figure 19. Prochaska et al. [151] explored raster mode and precession raster mode on a Zeeko IRP 100 to find which one is more suitable for the aspheric surface. The bonnet tool can remove the sub-surface damage caused by the previous process (e.g., grinding) in raster mode. However, because the tool kinematics leave a unique raster-scan structure, there is no reduction in roughness. In precession raster mode, the roughness was reduced by a factor of five because less mid-spatial frequencies were generated. So, the precession raster mode is more suitable for polishing non-planar workpiece surfaces.

#### 2.4.4. Characteristics

BP has been used in a variety of applications for its two apparent merits, i.e., high shape adaptability and controllability. The inflated bonnet provides a controllable elastic coefficient that enables the tool to adjust to different workpiece surface shapes [152]. Thus, making it suitable for aspheres [134] and freeform [153,154] components. Furthermore, BP allows the position of the bonnet, the angle of the polishing tool axis, the internal pressure, and the applied pressure of the bonnet to be adjusted according to the surface shape, making automatic control easier. The roughness realized by BP for different materials is illustrated in Table 6.

However, BP has challenges to realize sub-nanometer roughness for the following reasons:

(1) Polyurethane is a common polishing cloth in BP, which is extremely easy to wear during polishing. Therefore, the material removal function may not be constant.
(2) Due to the poor rigidity of the soft polishing tool, mid-spatial frequencies are generated by process kinematics [151], limiting the realization of the sub-nanometer roughness.
(3) The BP may be not the optimal choice when polishing small components [136] or workpieces with complex optical surfaces. This is because the bulged bonnet probably interferes with the workpiece surface.

### 2.5. Discussion on the Chemical Modification Polishing Approaches

This chapter provides an overview of what the authors have classed as chemical modification polishing techniques. In CMP, BP, and PAP, the workpiece surface is first modified and then removed with abrasives harder than the modified layer and softer than the bulk material. To be specific, CMP and BP use chemical modification and can be applied to ceramics, glass, metals, semiconductors, etc. PAP uses plasma to irradiate materials such as SiC, GaN, SCD, etc. CARE mostly uses catalysts to assist in etching SiC. The surface atoms of the workpiece are modified and removed by the etchant simultaneously.

Chemical modification polishing approaches allow a good balance between processing accuracy and costs. During CMP, PAP, and CARE, the rotating spindle of the workpiece misaligns with that of the polishing pad, enabling a multidirectional kinematic trajectory and uniform material removal. As a result, the simple structure can achieve sub-nanometer roughness when polishing planar surfaces.

Chemical modification polishing is not optimal for curved surfaces. BP draws on the chemical modification in the CMP approach to polish curved surfaces. The roughness cannot reach ACS precision because there is no multidirectional kinematic trajectory to remove the imprint left by mechanical forces in BP. The literature survey shows that no research has been found on applying PAP and CARE to curved surfaces. Approaches to generate a Gaussian removal function and to plan tool path are the gaps for future research.

In addition, there are electrochemical modification [158], anodic oxidation [109], and ultraviolet photocatalysis modification [159] that can be considered for polishing. The search for more suitable modification approaches and finding a balance between modification and removal can focus research to improve the efficiency and precision of the chemical modification polishing processes.

## 3. Non-Modification Polishing Approaches

### 3.1. Elastic Emission Machining

Elastic emission machining (EEM) was proposed by Mori in 1976 [160], which is an ultra-smooth and noncontact polishing process that enables atomic-scale precision [161].

#### 3.1.1. Removal Mechanism

**Figure 20:**
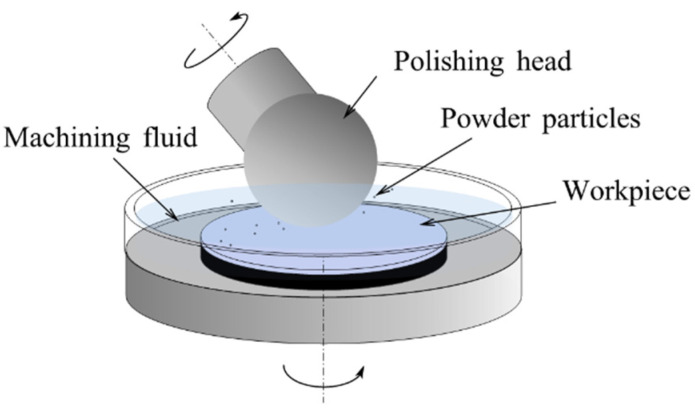
The schematic diagram of elastic emission machining [30].

In EEM, fine powder particles are brought to the workpiece surface by a flow of machining fluid through the spinning of a polishing head, and impact the workpiece surface at a small incidence angle [161], as shown in Figure 20. A weak chemical reaction between the workpiece and the particles removes atoms from the workpiece. Because the mechanical interaction of the powder particles and the polishing head do not contribute to the workpiece surface, atomic-scale smooth surfaces without crystallographic damage can be achieved.

**Figure 21:**
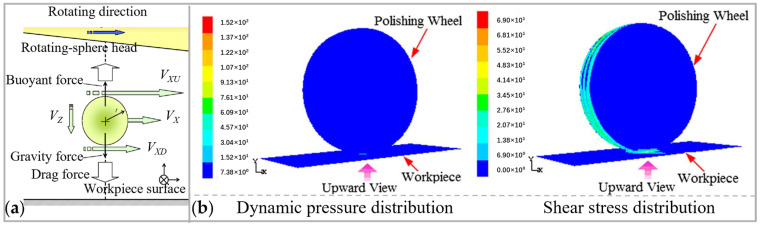
The material removal mechanism in elastic emission machining: (**a**) Schematic diagram of hydrodynamic effect [30]; (**b**) Distribution of dynamic pressure and shear stress under different clearances between the workpiece and polishing head [167].

The material removal mechanism of EEM has been studied from the point of view of hydrodynamic effects and physio-chemical reactions. Figure 21a illustrates the hydrodynamic effect in EEM [30]. The hydrodynamic effect generates an incompressible fluid film between the polishing head and the workpiece [162,163]. The thickness of this fluid film increases with the rotation speed of the polishing head [164]. Molecular dynamics simulations showed that when the powder partials are subjected to fluid shear, they take the first layer of atoms from the surface of the optical element [165,166]. Accordingly, hydrodynamic effects influence material removal by affecting the distribution and magnitude of dynamic pressure and shear stress [167], as shown in Figure 21b.

Material removal in EEM is promoted chemically, and there are two pieces of evidence to prove this. The first one is that the kinetic energy of the flowing particles is two orders of magnitude lower than the binding energy of the workpiece, indicating that the powder particles do not have sufficient energy to remove surface atoms physically [168]. The second one is the detection of the functional group Ce-O-Si on the polished cerium nanoparticles when polishing SiO_2_ with CeO_2_, from which the chemical impact reaction is validated [169].

**Figure 22:**
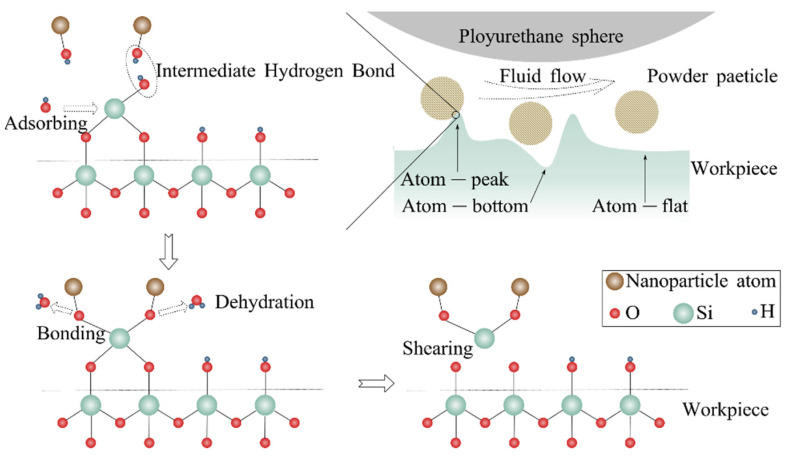
The interactions between workpiece surfaces of ultra-fine powders and works [165,167].

When the nanoparticles break the bonding between the workpiece surface and subsurface, chemical bonds of atoms at the surface peaks are more unsaturated than those at surface troughs, hence the chemical activity is higher at the peaks. Therefore, atoms on the micro protrusion are easier removed with nanoparticles, resulting in a sub-nanometer roughness on workpiece. A schematic of the chemical interaction process is shown in Figure 22 [167].

#### 3.1.2. Equipment Development

**Figure 23:**
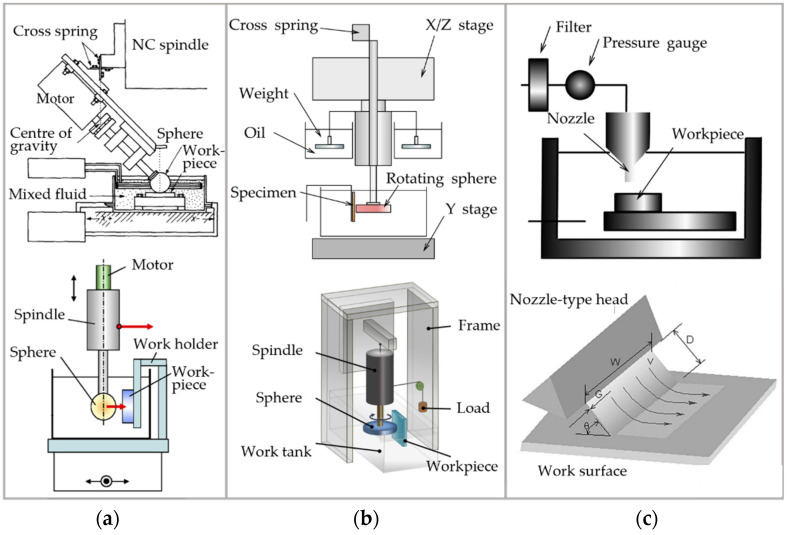
The schematic diagrams of elastic emission machining equipment: (**a**) Sphere type. Reproduced with permission from [30,162]; (**b**) Wheel type. Reproduced with permission from [170,171]; (**c**) Nozzle type. Reproduced with permission from [172,173]. EEM equipment is divided into clearance adaptive and clearance non-adaptive, depending on how the clearance between the sphere tool and the workpiece is controlled. In clearance adaptive equipment, the sphere or wheel can float above the workpiece automatically, because the slurry is continuously dragged into the narrow-converging polishing zone to produce sufficient pressure to lift the polishing tool [174]. In clearance non-adaptive equipment, the clearance is controlled by a numerically controlled motion system. The clearance in nozzle-typed equipment is adjusted by multi-axis motion system [173], indicating that nozzle-typed equipment is clearance non-adaptive. The clearance affects the size of the processing area [38], the incidence angle between the workpiece surface and machining fluid, and the machining efficiency. Therefore, the choice of the type of clearance control is a key factor to consider when developing EEM equipment.

Polishing head shapes can be divided into wheel, sphere, and nozzle. From the combinations of these, there are three types of EEM equipment. Systems using wheel and sphere polishing heads are similar. They consist of a rotating polishing head, a vessel filled with the polishing slurry, a numerically controlled multiply dimensional table as the feeding device, and an across-joint spring [162], as shown in Figure 23a,b. The polishing head is brought close to the workpiece surface by applying a load to the wheel or sphere [30]. The aperture size of the slit between the workpiece and the polishing head can be controlled by a cross-joint spring and the *z*-axis actuator [30].

In nozzle-typed EEM equipment, instead, a slurry flow is sprayed from a circle or square nozzle to the workpiece surface, as shown in Figure 23c.

#### 3.1.3. Expanding Application

In 1987, Mori et al. [162] showed a perfect crystalline state can be obtained by EEM, from the point of electron diffraction and transmission X-ray diffraction. Afterward, a great deal of research has been performed to expand the application of EEM.

**Figure 24:**
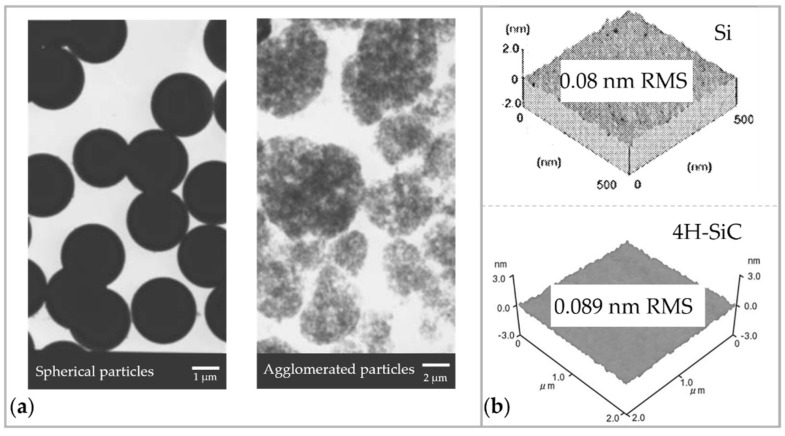
Application of elastic emission machining: (**a**) The TEM images of silica powder particles. Reproduced with permission from [175]; (**b**) The AFM images of different materials processed by elastic emission machining. Reproduced with permission from [172,176].

To enhance the material removal rate, agglomerated silica powder particles with larger surface area were used, as shown in Figure 24a. The removal rate was found to be enhanced 100 times more than the ordinary particles [175]. To evaluate the removal properties of different materials, Si [176], SiC [172], ULE, Zerodur [170], etc., were processed with EEM to achieve roughness below 0.1 nm in RMS, as shown in Figure 24b. To improve the process precision and efficiency, EEM was combined with PCVM [176] and numerically controlled plasma chemical vaporization machining [177] for fabricating the X-ray optics. Because PCVM can efficiently correct figure error with the spatial wavelength range from 1 to 10 mm, EEM can remove the residual figure error with the spatial wavelength close to 0.1 mm [177].

On the basis of the above-mentioned fundamental research, the industrial applications of EEM were also developed. In the field of optics, EEM could realize a spatial resolution of 100 μm in the manufacture of an X-ray mirror with the roughness of 0.52 in RMS [173]. In the same soft X-rays field, Hirata et al. [171] applied the rotating spherical EEM to a glass cylinder to produce an ellipsoidal mirror for soft X-ray microscopy, with the roughness of 0.16 nm in RMS.

#### 3.1.4. Characteristics

In EEM, the subsurface damage and surface scratches of the preprocessed surface are almost entirely removed. The processed surface is free of crystallographic disordered structure and plastic deformation [172], which is beneficial for polishing functional crystal materials. In addition, a submillimeter-size footprint with a stable removal rate can be created. Hence, it is suitable for polishing non-planar components with small curvature [171]. In terms of finishing capability, EEM can achieve atomic-level roughness, at 0.1 nm or even lower, as shown in Table 7.

Low processing efficiency is a significant problem in EEM. The reasons for the low material removal rate can be summarized as the following:

(1) There is a high frequency of collision between powder particles and between the powder particles and the polishing head. The energy is wasted by this ineffective collisional process.
(2) To achieve atomic-level removal, the thickness of the fluid film between the workpiece and polishing head needs to be tightly controlled at the micron scale to keep a small processing area. A too-small processing area causes an increase in polishing time.

### 3.2. Ion Beam Figuring

IBF was proposed and demonstrated by Wilson and McNeil in 1987 [180]. In their experiment, a so-called Kaufman ion source [181] was used to polish a fused silica optic to achieve a roughness of 0.55 nm in RMS, demonstrating the feasibility of the IBF [182]. IBF is a nonmechanical and noncontact figuring approach that can reach sub-nanometer precision by bombarding ions into the workpiece surface [183].

#### 3.2.1. Removal Mechanism

**Figure 25:**
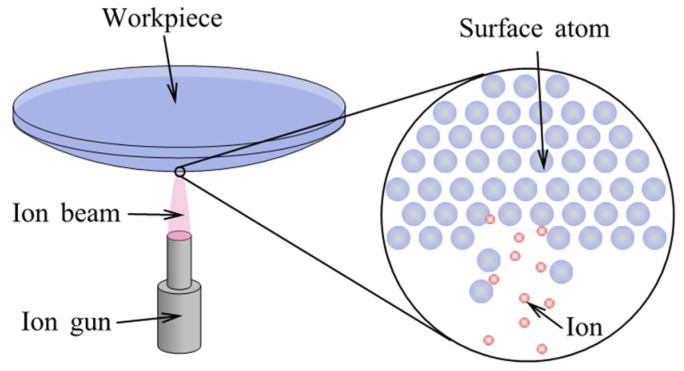
The schematic diagram of IBF [184].

Figure 25 shows the process principle of IBF. After the ion beam has bombarded the surface of the workpiece, the atoms that have gained energy continue to transfer energy to the surrounding atoms. When the energy gained by the collision is large enough to overcome the surface binding energy, atoms or atoms clusters will be removed from the workpiece surface, thus achieving atomic-level material removal [184]. The physical process to form sputtering atoms at the atomic scale is a collision process. The fraction of energy in a head-on collision is [185]:

(16) $$\gamma =\frac{E_{2}}{E_{0}}=\frac{4m_{1}m_{2}}{{\left(m_{1}+m_{2}\right)}^{2}}$$

where *γ* is the energy transfer factor, *E*_2_ is the kinetic energy transferred to the target particle, *E*_0_ is the initial kinetic energy of the projectile, and *m*_2_ and *m*_1_ are the mass of the projectile and target particle, respectively.

**Figure 26:**
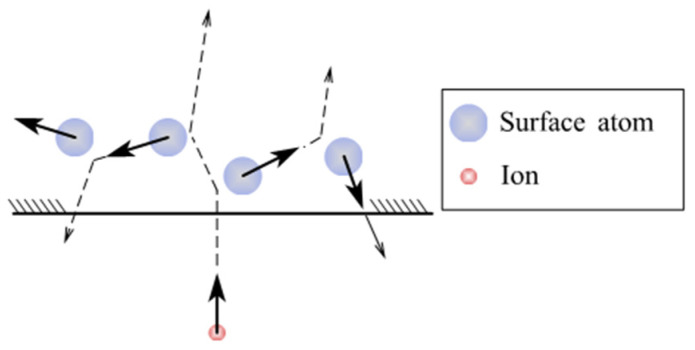
The series of collision processes [187].

How to improve the material removal rate and processing accuracy is the focus of the research on the removal mechanism of IBF. Meinel et al. [186] discovered in 1965 that when fused silica was exposed to an ion beam, uniform material removal occurred, but at a relatively low rate. Acceptable material removal rates were not available until 1977 [181] and are related to the sputtering mechanism. To be specific, ion cascades generated by direct impingement of the incoming ions dominate the sputtering mechanism for heavy ions [185]. This cascade may extend over a considerable region inside the target, increasing the material removal rate [187], as shown in Figure 26. The control of the incidence angle of ion beam directly affects whether IBF can obtain sub-nanometer roughness because the sputtering yield is dependent on the incidence angle [187].

#### 3.2.2. Equipment Development

**Figure 27:**
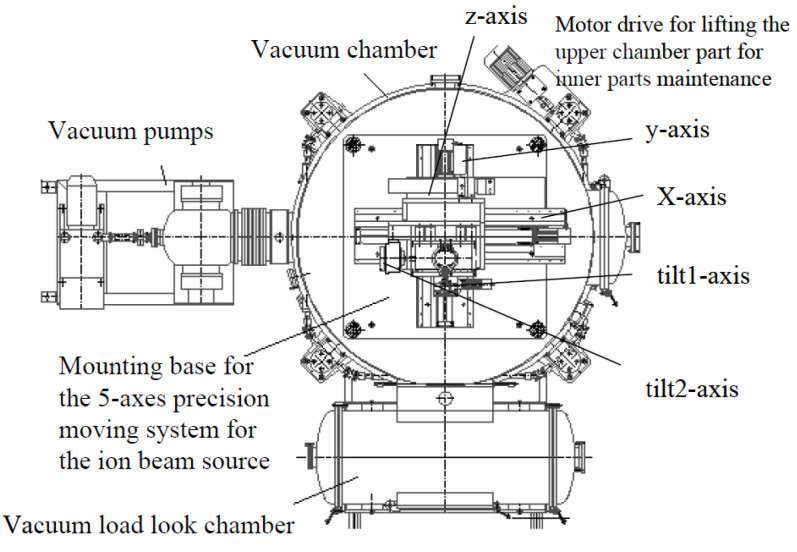
The IBF equipment developed by Nanotechnologie Leipzig GmbH [190].

The simple structure and the high machining accuracy is the distinguishing characteristic of IBF equipment. The University of Rochester and Eastman Kodak developed the first IBF processing system, which was successfully applied to the figuring of a 2.5 × 2.5 × 0.6 m mirror [188]. Marcel et al. [189] used a 3-axis movement system for carrying out sophisticated IBF. To improve the machining accuracy, a series of IBF machines with an advanced dwell time algorithm was developed by Nanotechnologie Leipzig GmbH [190], as shown in Figure 27.

#### 3.2.3. Effect of Original Roughness

**Figure 28:**
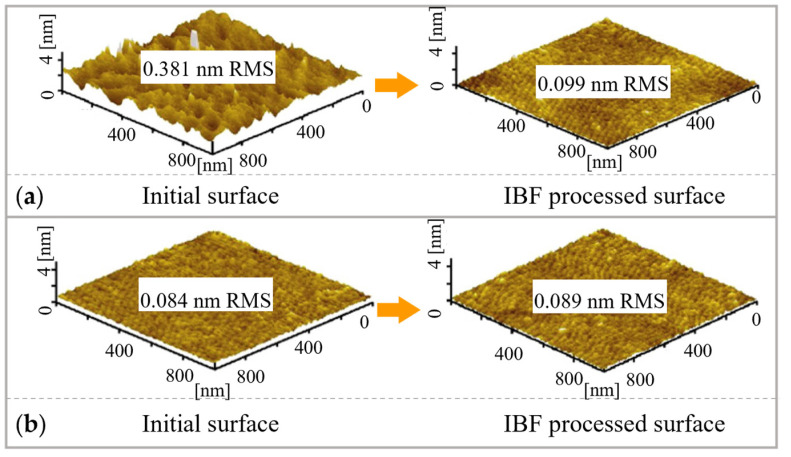
The AFM images of unprocessed and IBF-processed SCD. Reproduced with permission from [192]: (**a**) The first sample; (**b**) The second sample.

IBF can sometimes lead to the deterioration of roughness. Kamimura et al. [191] used scanning atomic force microscopy to investigate the original surface roughness and the results after IBF, showing that the surface roughness will be degraded if the ion voltage is too intense. Mahmud et al. [192] performed IBF on SCD with initial surface roughness of 0.381 nm in RMS and 0.084 nm in RMS, indicating that a rough surface becomes smooth, and a smooth surface becomes rough, following a converging trend where the roughness saturates at a particular level, as shown in Figure 28.

#### 3.2.4. Characteristics

The removal function in IBF is “sub-aperture”, i.e., significantly smaller than the size of the optics. This facilitates IBF in polishing non-planar workpieces (aspheric [190,193], shallow spherical, parabolic [36], and non-axially symmetric [194]) of different materials (ceramics, stainless steel, magnesium alloy, high-speed steels, BK7 [195], and SiC [196]). In addition to surface shape adaptability, the IBP process has high stability, repeatability, and accuracy, as shown in Table 8. This is because IBF can remove material from the workpiece surface at the atomic level, is not affected by wear, and there is no edge roll-down effect [196].

Although the precision of IBF is high, this approach also has the following flaws:

(1) The aperture of the workpieces is limited by the size of the essential vacuum chamber.
(2) The material sputtering phenomenon ensures the atomic-level material removal, resulting in the difficulty of improving the machining efficiency and the limitation of brittle materials with a high coefficient of expansion.
(3) The requirements for the original roughness of the workpiece make IBF costly [24].

These drawbacks have limited the widespread use of IBF. Currently, IBF is only suitable for the final stage of optical machining where very high machining accuracy is required.

### 3.3. Magnetorheological Finishing

MRF was invented by Kordonski in 1988 [202]. Due to the characteristics of high precision and low surface defects, MRF is, as of today, considered the state-of-the-art commercial figuring technology.

#### 3.3.1. Removal Mechanism

**Figure 29:**
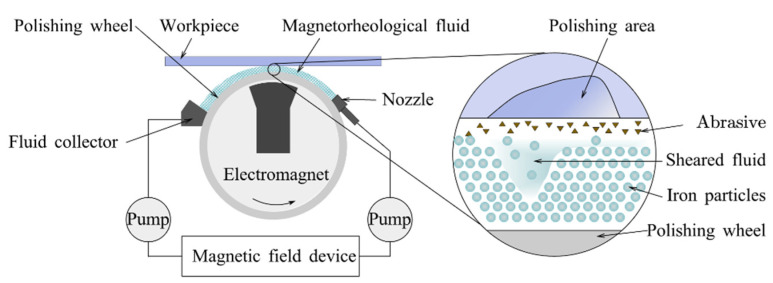
The schematic diagram of MRF [203].

Figure 29 shows the schematic of MRF. The wheel-typed polishing tool delivers the magnetorheological fluid mixed with abrasive particles to the surface of the workpiece. When the magnetorheological fluid moves with the wheel near the converging gap formed by the workpiece and the wheel, the magnetic gradient field causes the polishing fluid to agglomerate. The magnetorheological fluid creates a unique pressure distribution in the converging gap and exerts a shear force, making it a material removal zone [203].

The magnetic gradient field generates agglomeration of the polishing fluid and high yield stress in the converging gap, exerting a shear force to remove material [203]. The magnetorheological fluid is compressed after a magnetic field is applied. The static yield stress can reach as high as ten times the yield stress without compression. Tang et al. [204] examined the microstructure of the magnetorheological fluid before and after compression. Without compression, the microstructure was dominated by a single chain of magnetic particles. After the compression, the single chain structure turns into thicker columns with a thickness of 50 μm, thus the yield stress was enhanced.

When applying the magnetic field, the interparticle forces are calculated numerically by considering the magnetostatics between the particles inside the aggregates. The total dipole moment per unit volume can be calculated [205]:

(17) $$\frac{\alpha -1}{\alpha +2}\alpha^{3}H=C\cdot m$$

where *α* is the radius of the particles, **H** is the average field inside the unit cell, **C** is a matrix that depends on the relative positions of the particles inside the unit cell, and **m** is the vector defining the dipole moment of each particle.

Therefore, the size and density of the abrasive particles and the strength of the magnetic field determine the yield stress of the magnetorheological fluid in the convergence gap.

#### 3.3.2. Equipment Development

**Figure 30:**
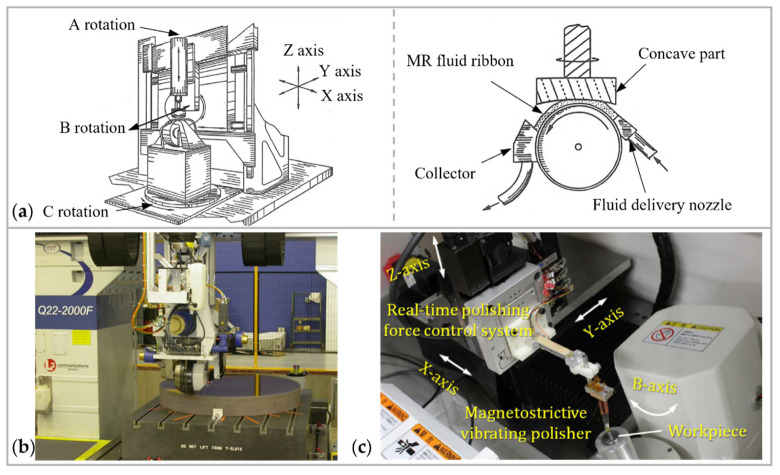
The equipment development of MRF: (**a**) The machine with a vertical wheel-type polishing head [214]; (**b**) Q22-2000F machine [215]; (**c**) The vibration-assisted system [212].

The structure of MRF equipment is constantly evolving to achieve lower roughness, as shown in Figure 30. In 1994, a team led by Kordonski operated a pre-prototype MRF machine [202,206,207]. In this phase, a trough was utilized to extrude a ribbon of magnetorheological fluid into the magnetic field. In 1996, the OptiPro company constructed an MRF machine with a vertical wheel-type polishing head to replace the trough, allowing for aspheric optical polishing [208]. In 1997, the QED company was established and produced the first commercial equipment for MRF, the Q22, which officially commercialized MRF [209]. In 2011, Guo et al. [210,211] developed a vibration-assisted polishing system by using magnetostrictive material: surface roughness was improved to 0.5 nm in RMS [212] and 0.4 nm in Ra [213] for micro-aspheric glass lenses.

#### 3.3.3. Magnetorheological Fluid Development

**Figure 31:**
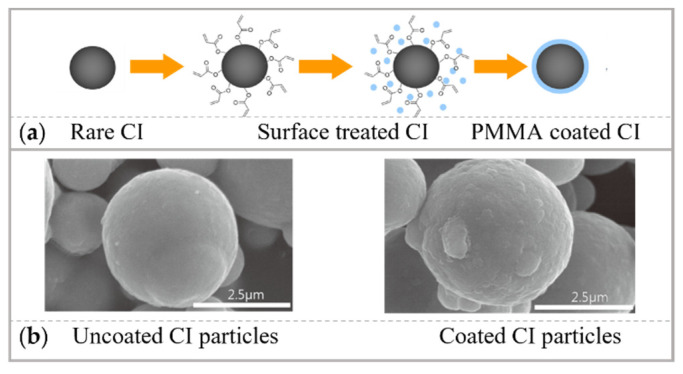
The magnetorheological fluid development: (**a**) The schematic diagram of the PMMA coating process. Reproduced with permission from [218]; (**b**) The SEM images of uncoated and coated CI particles. Reproduced with permission from [216].

The reduction in both roughness and defectivity can be achieved by choosing the proper magnetorheological fluid. To polish potassium dihydrogen phosphate crystals, the oil-based magnetorheological fluid is a suitable option. In fact, as the potassium dihydrogen phosphate crystals are extremely soluble in water, the magnetorheological fluid carrier liquid must be nonaqueous [216]. For polishing fused silica, alkaline additives in the magnetorheological fluid can achieve a higher material removal rate and finishing quality simultaneously, due to the zeta potential adjustment generated by the pH enhancement of magnetorheological fluid [217]. When polishing BK7 optical glass, the soft magnetic carbonyl iron (CI) particles were coated with polymethyl methacrylate (PMMA). Roughness 0.86 nm in Ra was obtained after overcoming the conventional corrosion problem that occurs when CI particles were used. PMMA coating process and uncoated/coated CI particles are shown in Figure 31a,b [218]. In addition, to avoid sedimentation problems in conventional MRF, magnetorheological elastomers based on very high kinematic accuracy were created [219,220,221].

QED offers a stable, high-performance magnetorheological fluids family, including D10, C10+, D11, C12, and D20, which can meet the polishing requirements of poly-crystalline materials (tungsten carbide and silicon carbide), hardest materials (sapphire), and smoothing of diamond turning marks on infrared optics [222]. A novel magnetorheological fluid, C30, was designed to reduce defects, increase the laser optics damage threshold, and improve the roughness to 0.1 nm in RMS on a variety of materials, such as SiO_2_ [223], CaF_2_, and Ni [224].

In addition to polishing, anisotropic magnetorheological fluids have other industrial applications [225]. For example, Ford company motor has patented automotive bushings based on magnetorheological elastomers, due to the mechanical anisotropy of magnetorheological elastomers that can be used to minimize the strength of elastomeric bearings [226]. Meanwhile, magnetorheological foams are used as dampers. Most of the stress is taken up by the arrangement of the field-induced magnetizable particles in the magnetorheological fluid, so that wear of the magnetorheological foam rarely occurs and therefore dampers based on magneto-resonant foams have a longer life [227].

#### 3.3.4. Characteristics

There is no wear on the polishing tool in MRF, since the magnetorheological fluid is constantly reconditioned to maintain the viscosity and temperature of the fluid [228]. Furthermore, the removal function has a high peak removal rate and a small area of action, making it appropriate for the removal of medium and high-frequency residuals in components [216]. The ability to polish a wide range of surface shapes (aspheric [229], concave [230], freeform [231], planar, sphere, cylinder, and square [232]) and different materials (Cu [233], SiO_2_ [234], and Si [235]) with high accuracy greatly increases the commercial impact of MRF, as shown in Table 9.

The reliance on magnetic fields and the converging gap makes MRF make MRF suffer from the following problems:

(1) MRF equipment with special magnetorheological fluid requires a suitable gradient magnetic field generator and high accuracy of the kinematic system, resulting in high cost.
(2) During the MRF process, there is 1~2 mm clearance between the large polishing wheel and the workpiece. When polishing concave surfaces with a small radius of curvature and the internal surface of the workpiece, collisions may occur. This small converging gap limits the curvature and complexity of the workpiece.
(3) MRF cannot be applied to ferromagnetic materials. Due to the presence of the electromagnet, the ferromagnetic workpiece may interrupt the processing stability.
(4) The sedimentation of micron-sized magnetic powder is a common problem, which is the key issue limiting the prolonged utilization in MRF [239].

### 3.4. Fluid Jet Polishing

FJP was investigated by Fähnle [240] in 1998. FJP is a sub-aperture polishing technique that uses the abrasive slurry jet to remove the material of the workpiece surface in brittle materials [241,242].

#### 3.4.1. Removal Mechanism

**Figure 32:**
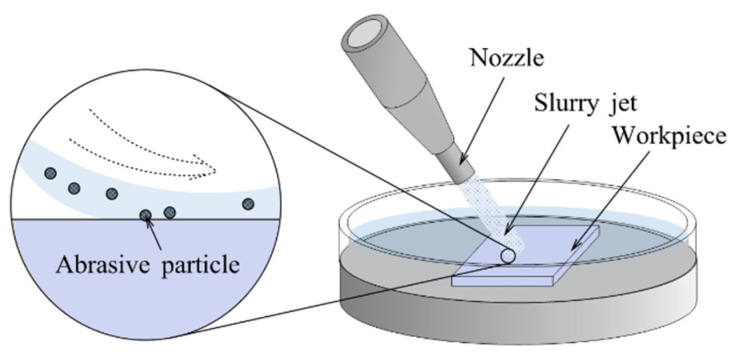
The schematic diagram of FJP [244].

A low-pressure water beam with abrasive particles is referred to as the slurry jet, which is accelerated through a nozzle located above the workpiece surface. The slurry jet, at a certain angle, impacts the workpiece at high speed to remove material and can be reused after collection [243], as shown in Figure 32.

When the abrasive particles impact the workpiece, erosion phenomena occur on the workpiece surface. The amount of material removed from the workpiece surface determines whether the FJP can achieve sub-nanometer roughness. Bitter [245,246] proposed that there are two types of wear in erosion to solve the amount of material removed. One is deformation wear caused by repeated deformation during collisions, the other is cutting wear caused by the cutting action of the free-moving particles. The total wear *W* expressed in units volume loss is:

(18) $$W=W_{D}+W_{C}=\frac{M{\left(V\sin\alpha -K\right)}^{2}}{2\varepsilon }+W_{C}$$

where *W_D_* is deformation wear in units volume loss, *M* and *V* are total mass and velocity of impinging particles, respectively, *α* is impact angle, *K* is a constant, and *ε is* the energy needed to remove a unit volume of material from the body surface, and *W_C_* is cutting wear in erosion, that can be calculated by the cutting mechanism [247]:

(19) $$W_{C}\approx \frac{MV}{P}f\left(\alpha \right)$$

where *P* is the horizontal component of flow pressure between the particle and the surface, and *f*(*α*) is a function of the angle of impact measured from the surface to the particle velocity vector.

**Figure 33:**
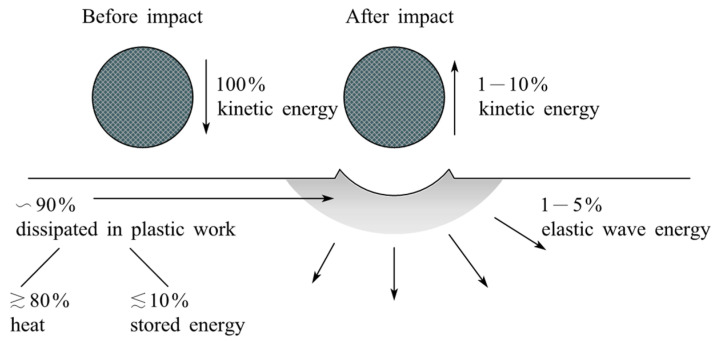
The energy balance before and after the normal impact of a spherical erosive particle [248].

To obtain *ε*, the criterion of critical plastic strain needs to be considered, since at least 90% of the initial kinetic energy of the particle is dissipated after normal impact, as shown in Figure 33. Therefore, it is permissible to ignore elastic effects when calculating the mass loss from the target per unit mass of impinging particles *E* [248]:

(20) $$E=0.033\frac{\alpha \rho \sigma^{1/2}V^{3}}{\varepsilon_{c}^{2}P^{3/2}}$$

where *α* is the fraction of the volume of the indentation, *ρ* is the density of target material, *σ* is the density of eroding particles *ε_c_* is erosion ductility.

According to Equations (18) to (20), the key parameters for determining the roughness of the workpiece are the angle between the nozzle and the workpiece, the impact pressure of the jet, and the density of the abrasive particles.

#### 3.4.2. Development of Roughness Reduction

**Figure 34:**
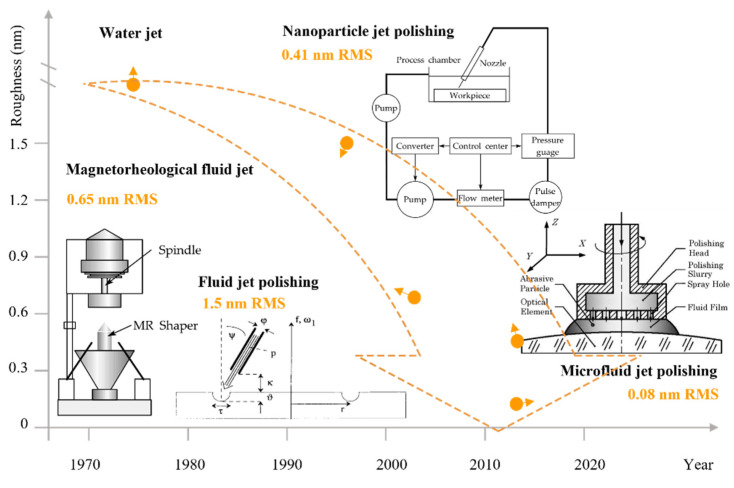
The historical development of FJP. Reproduced with permission from [240,249,250,251,252].

After decades of development, the roughness that can be achieved by FJP continues to decrease, as shown in Figure 34. In 1978, water jet was applied to the logging industry. It was discovered that adding abrasive particles to a pure water jet increased the cutting efficiency [249]. During this time, it was only used to cut wood without roughness requirements. In 1998, Fähnle et al. [240] lowered the pressure of a fluid jet with abrasives, investigated the micro-removal effect on workpiece surfaces, and introduced fluid jet technology in the polishing field.

To decrease the roughness of FJP, a series of related jet precision machining techniques such as magnetorheological fluid jet [250], nanoparticle jet polishing [252], and microfluid jet polishing [251] have been proposed based on FJP. In 2003, Kordonski et al. [250] demonstrated that the impingement of magnetorheological fluid with the abrasive particle is stabilized compared with normal FJP slurry, resulting in high precision surfaces with angstrom level roughness.

In 2013, Peng et al. [252] presented nanoparticle jet polishing that decreased the SiO_2_ surface roughness to 0.41 nm in RMS. The number of hydroxyl groups on a convex workpiece surface is usually larger than that of a concave surface. During the polishing, the chemical reaction between nanoparticles and surface atoms is easily activated, thus allowing the workpiece to achieve a sub-nanometer roughness. Ma et al. [251] proposed microfluid jet polishing in 2013. This approach decreased high-spatial frequency surface roughness to 0.08 nm in RMS for fused silica. In the microfluid jet polishing process, the polishing fluid flows out from the polishing toll, and the dynamic pressure caused by the polishing slurry motion lifts the polishing tool. Chemical reactions between the workpiece and the fine abrasive particles caused the topmost atoms on the workpiece surface to be removed and atomic finishing was achieved.

#### 3.4.3. Gaussian Removal Function

**Figure 35:**
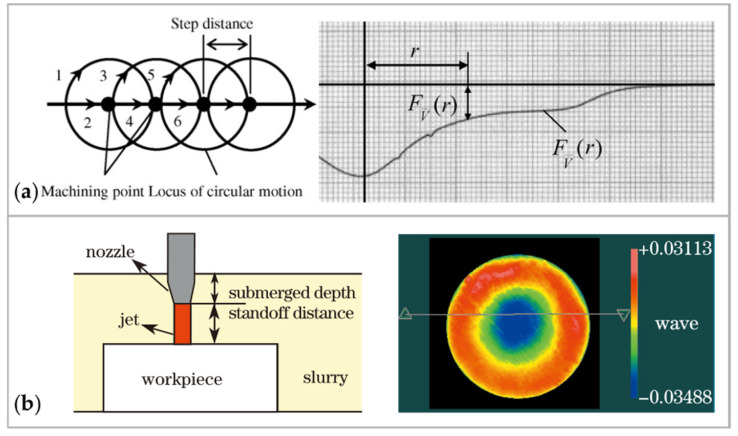
The FJP setup and Gaussian removal function: (**a**) Circular motion system. Reproduced with permission from [253]; (**b**) Submerged jet polishing [257].

In FJP, the removal function is W-shaped when the slurry jet strikes the workpiece surface vertically [253]. However, a Gaussian removal function is a preferred type, since the Gaussian removal function is appropriate in iterative manufacture to achieve form accuracy [254]. Horiuchi et al. [253] introduced a circular motion to produce a preferable removal function with an axis-symmetric V-shape. The circular motion is performed around every machining point distributed with a constant step on the scanning path, as shown in Figure 35a. Fang et al. [255] proposed a method for multi-position synthetic jetting. In this method, an ideal Gaussian removal function is generated when the slurry jet impacts the workpiece obliquely from four positions. Yu et al. [256] designed an air-driving FJP system, where the slurry is guided to mix with airflow inside the nozzle cavity to produce the Gaussian removal shape. Shi et al. [257] reported submerged jet polishing. Gaussian shape removal function can be obtained by submerging the nozzle and workpiece in liquid, as shown in Figure 35b.

#### 3.4.4. Characteristics

The removal by FJP can take place over small areas [258]. The polishing tool in FJP is a jet beam with low pressure and the beam diameter depends on the nozzle outlet diameter, so polishing small structures can be carried out even for highly steep concave parts and a variety of cavities [250]. These advantages make FJP an attractive solution for optical surface finishing. On different types of glass, roughness values between 0.08 to 0.5 nm can be achieved with FJP, as shown in Table 10.

The removal function generated by the slurry jet is difficult to maintain constant due to factors such as polishing slurry delivery pressure, flow rate, and air disturbance. This is a challenge that usually needs to be considered during the FJP process.

### 3.5. Discussion on “Non-Modification” Polishing Approaches

#### 3.5.1. Characteristics

The distinguishing feature of “non-modification” polishing approaches is that the workpiece surface is not modified prior or during the process and the material removal medium (abrasive particles, ion beam, magnetorheological fluid, water jet) removes the surface atoms directly, sub-nanometer roughness to be achieved with little or no mechanical force. As sub-aperture tools, the described non-modification polishing approaches are highly flexible when it comes to freeform geometries [261], allowing the machining of a wide range of non-planar surfaces such as spherical and aspherical. However, these polishing approaches remove surface material at an atomic scale, resulting in lower material removal rates compared to chemical modification polishing approaches.

**Figure 36:**
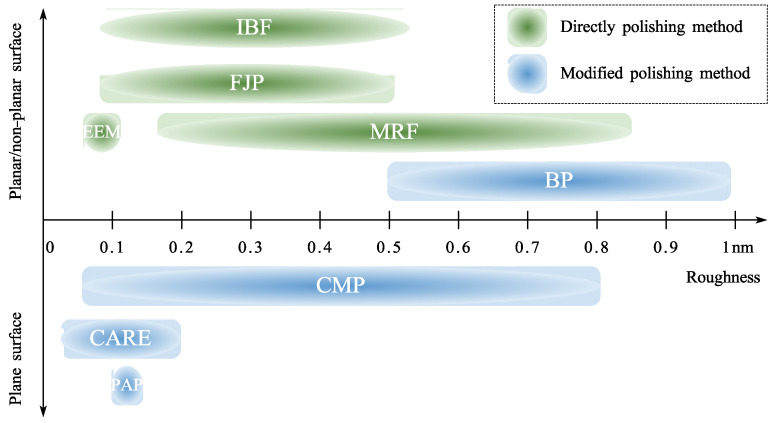
The roughness range of different machining approaches.

Figure 36 describes the roughness that can be achieved by the different polishing approaches. Chemical modification polishing methods can achieve sub-nanometer roughness on planar workpieces, but the roughness is higher when polishing non-planar workpieces, due to mechanical forces producing scratches on the workpiece surface. When processing planar surfaces, the workpiece and the polishing pad will rotate on their respective spindles off-axis to each other, thus smoothing out repetitive marks. When processing non-planar workpieces, the removal function travels over the workpiece like the footprint, making it difficult to completely remove the scratches.

#### 3.5.2. Processing Chain for Non-Planar Surface

**Figure 37:**
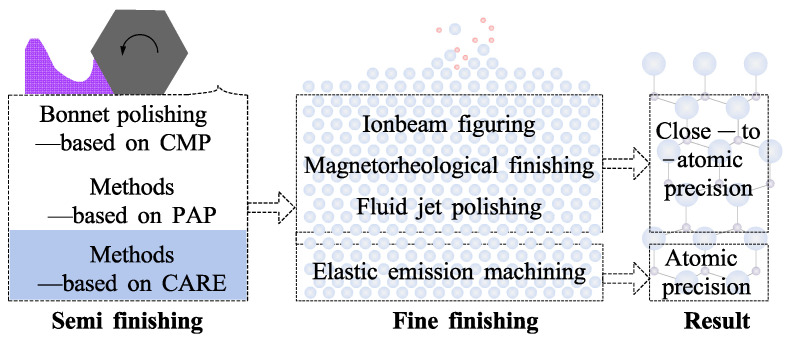
The processing chain for non-planar surface.

A possible processing chain is proposed for non-planar surfaces to efficiently achieve sub-nanometer roughness, as shown in Figure 37. The first step would be to semi-finish the lapped workpiece using a chemical modification polishing approach, which is expected to efficiently achieve the required roughness at a sub-nanometer scale. However, sub-surface damage would be inevitably introduced when the small polishing tools contact the surface. The second step would be a fine finish using a non-modification polishing approach, which is expected to further remove atoms from the top surface. These non-contact machining processes may have a potential to remove the sub-surface damage to achieve very high surface quality, meeting the roughness requirements of 0.2 nm and 0.15 nm in RMS for hard X-ray and extreme UV systems, respectively.

## 4. Conclusions and Perspective

When ultra-precision polishing was introduced at early stages, the mechanisms were unclear, the material removal rates unacceptable, and the equipment not sophisticated [262]. The polishing approaches reviewed in this article present the typical processing technologies available to achieve surface roughness in an Ångström level. Material removal mechanisms, equipment development, and industrial applications of such methods have been reviewed in detail with the aim of developing a process chain capable of deterministically producing Ångström level roughness. In general, chemical modification polishing approaches achieve high machining precision on planar workpieces and relatively low accuracy on non-planar surfaces. Non-modification polishing approaches have shown their capability to obtain surface roughness in Ångström level on planar and non-planar workpieces, though research are still required to improve polishing efficiency. Polishing is a main approach to realize atomically precise manufacturing. To achieve better roughness, efficiency, and eco-friendly processes when polishing diverse materials with different surface geometries, the perspectives can be presented as the following:

(1) Limited range of processing materials

Polishing methods, including CMP, BP, and PAP have demonstrated their capability of achieving surface roughness at ACS. These methods involve first modifying the surface material to a softer state before removal, achieving a delicate balance between precision and cost-effectiveness. To broaden the range of materials that can be processed at such high precision, incorporating techniques such as electrochemical modification, anodic oxidation, ultraviolet photocatalytic modification, and thermal oxidation into the polishing process is worth considering. Currently, CARE is primarily utilized for polishing SiC, but the range of materials that can be polished with CARE would be greatly extended by finding suitable catalysts for other materials. Therefore, this method has great potential for industrial applications.

(2) Limited range of workpiece shapes

In chemical modification polishing approaches, CMP, PAP, and CARE can effectively reduce surface roughness to 0.2 nm in Ra or RMS on planar workpieces by utilizing planar disc-shaped polishing pads. However, these methods are currently not suitable for non-planar surfaces due to the limitations of the surface shape of the polishing pad. One solution to this problem is to modify the flat polishing pad to a smaller, curved polishing head or to use elastic compliance tools [263,264] in combination with computer-controlled optical surfacing technology (Computer-controlled optical surfacing technology are described in detail in Appendix A). For example, by investigating the relationship between temperature, radio frequency power, and material removal rate in PAP with a curved polishing head, it is possible to calculate the entire surface morphology by convolving the material removal rate and dwell time at a single point, providing a foundation for non-planar polishing.

(3) High processing costs

Non-modification polishing approaches tend to have higher processing costs compared to chemical modification polishing approaches. However, they may be the main way to achieve ACS polishing on non-planar workpieces. Hence, it is important to reduce the cost and increase the efficiency in non-modification polishing. For example, in MRF, the service life and efficiency of magnetorheological fluids can be enhanced by adding additives such as anti-settling agents and co-dispersants to improve the stability of magnetorheological fluids, thus extending the service life and solving the in-use-thickening problem. Additionally, studying the relationship between temperature and material removal rate in MRF can make the process more efficient. Addressing the in-use-thickening problem [265] of magneto-rheological fluids will allow for more effective MRF.

(4) Environmental pollution

Chemical reagents are a common necessity in polishing methods, such as HF, which is typically utilized to remove the contamination caused by slurry in CMP and as an etchant in CARE. However, HF is an environmental pollutant, and a highly hazardous substance, resulting in significant costs for subsequent recovery and disposal. The plasma-related process is an emerging technology with wide application prospects in polishing, but the impact of plasma on the workpiece itself still requires further studies. Researchers are currently investigating the use of water instead of HF in CARE, but current material removal rate is low, and improving material removal rates based on water is also a promising future research direction.

## Figures and Tables

**Table 1:** The ultimate roughness achieved with CMP for different materials.

Material	Roughness (nm)	Year
Glass	Ra 0.8	2010 [85]
GaN	Ra 0.18	2012 [86]
SCD	Ra 0.09	2014 [84]
SiC	Ra 0.05	2014 [87]
Sapphire	Ra 0.065	2015 [88]
SiO_2_	Ra 0.193	2019 [89]
YAG	Sa 0.45	2019 [61]
Oxide silicon	RMS 0.15	2020 [90]

**Table 2:** The processing parameter for different materials using PAP.

Material	SiC [94]	GaN [99]	SCD [100]
Modified material	SiO_2_	GaF_3_	~
Process gas	He, 1.7% H_2_O	He, CF_4_	Ar, 0.67% H_2_O
Radicals	OH molecules	F molecule	OH molecules
Flow rate (L/min)	1.5	~	0.1
RF power (W)	6	18	~
Abrasive	CeO_2_ (*Φ* 0.5 μm)	CeO_2_ (*Φ* 1.2 μm)	~

**Table 3:** The ultimate roughness achieved with PAP for different materials.

Material	Roughness (nm)	Year
RS-SiC	Ra 0.48	2013 [109]
4H-SiC	RMS 0.1	2013 [97]
GaN	Sq 0.1	2015 [99]
CVD-SiC	RMS 0.69	2017 [102]
SCD	Sq 0.13	2018 [100]

**Table 5:** The ultimate roughness achieved with CARE.

Material	Roughness (nm)	Year
On-axis 4H-SiC (0001)	RMS 0.093	2007 [115]
GaN	RMS 0.198	2008 [117]
8° off-axis 4H-SiC (0001)	RMS 0.044	2008 [119]
4° off-axis 4H-SiC (0001)	RMS 0.051	2017 [123]

**Table 6:** The ultimate roughness achieved with BP for different materials.

Material	Roughness (nm)	Year
BK7	Ra 0.5	2002 [141]
Nickel-coated aluminum	Ra 1	2003 [155]
Stavax stainless steel	Ra 1	2003 [155]
Electroless nickel	Ra 0.316	2019 [156]
Aluminum alloy	RMS 0.58	2019 [157]

**Table 7:** The ultimate roughness achieved with EEM for different materials.

Material	Roughness (nm)	Year
SiC	RMS 0.089	2005 [172]
Zerodur	RMS 0.116	2007 [170]
Si	RMS 0.050	2012 [168]
Quartz glass	RMS 0.080	2015 [178]
Monocrystalline Si	RMS 0.151	2022 [179]

**Table 8:** The ultimate roughness achieved with IBF for different materials.

Material	Roughness (nm)	Year
Fused silica	RMS 0.08	2001 [197]
GaSb	RMS 0.18	2003 [198]
SiC	Rq 0.54	2010 [199]
SCD	RMS 0.1	2012 [192]
ULE	RMS 0.18	2014 [200]
Si	RMS 0.5	2022 [201]

**Table 9:** The ultimate roughness achieved with MRF for different materials.

Material	Roughness (nm)	Year
Si	RMS 0.25	2004 [235]
Metals	RMS < 1	2006 [236]
Cu	Ra 0.102	2012 [233]
SiO_2_	Ra 0.167	2013 [234]
BK7	Ra 0.86	2015 [218]
Nickel	Ra 0.30	2016 [237]
NEXCERA	RMS 0.61	2017 [238]

**Table 10:** The ultimate roughness achieved with FJP for different materials.

Material	Roughness (nm)	Year
K9	Ra 0.519	2009 [244]
Quartz glass	RMS 0.225	2013 [259]
SiO_2_	RMS 0.410	2013 [252]
Fused silica	RMS 0.080	2013 [251]
Electroless nickel	RMS 0.280	2013 [260]

**Figure A1:**
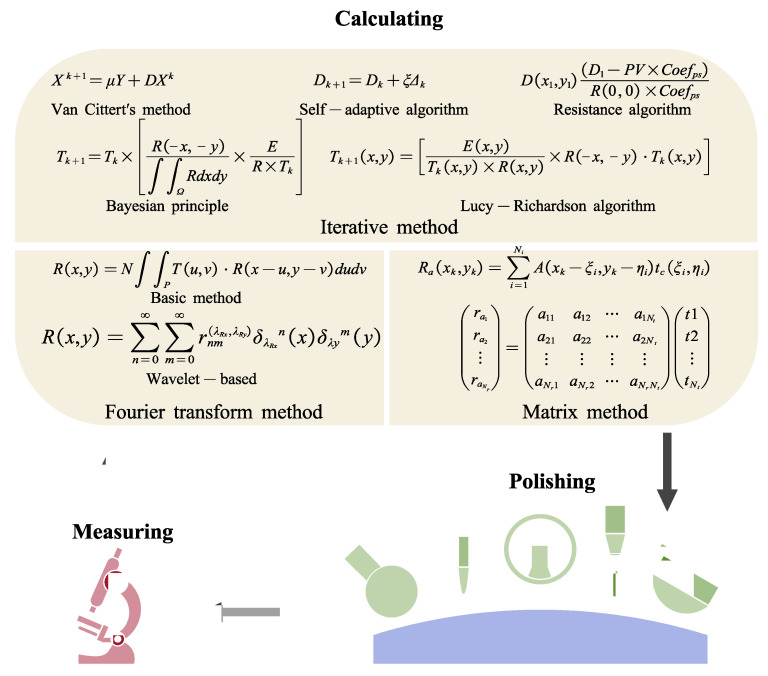
The process flow of the polishing workpiece using computer-controlled optical surfacing.

**Figure A2:**
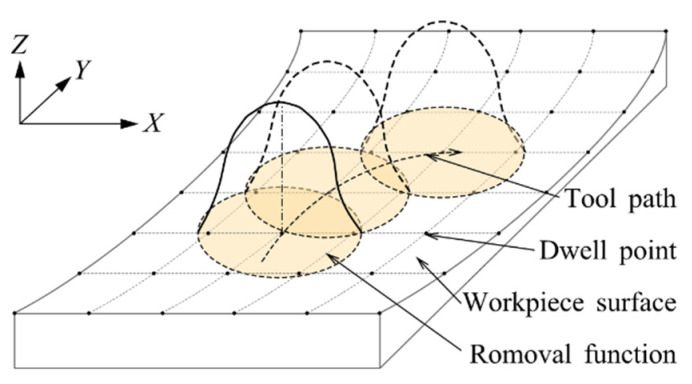
The schematic diagram of the material removal process of CCOS [253].

## Data Availability

No new data were created or analyzed in this study. Data sharing is not applicable to this article.

## Appendix A

Algorithms of the dwell function for BP, EEM, IBF, MRF, and FJP will be discussed in this appendix.

The computer-controlled optical surfacing (CCOS) uses a polishing pad whose size is much smaller in diameter than the workpiece to polish the workpiece under computer control [266]. BP, EEM, IBF, MRF, and FJP belong to CCOS. The process flow of CCOS is to measure the workpiece shape, then calculate the tool path and dwell function, and finally polish the workpiece, as shown in Figure A1. This process is repeated until the form accuracy and roughness are up to the requirement.

Figure A2 shows the CCOS polishing process in detail. The polishing head polishes point by point along the planned tool path on the workpiece surface. The envelope line of the removal function forms the final workpiece surface profile [267].

The material removal amount in CCOS is a convolution between the removal function and the dwell function, so the determination of the dwell function is the deconvolution process. Three algorithms can solve the dwell function: Fourier transform method, iterative method, and matrix method [266].

(1) In the Fourier transform method, the material removal amount and the material removal function are first Fourier transformed. Then, the Fourier transform of material removal amount was divided by that of the material removal function to obtain that of the dwell function. The dwell function can be calculated by inverse. The tool removal profile could be represented by the following equation [268]:

(A1) $$R\left(x,y\right)=N\int \int_{P}T\left(u,v\right)\cdot R\left(x-u,y-v\right)dudv$$

where *T*(*x*,*y*) is the dwell time function, *P*(*x*,*y*) is the path of the tool over the workpiece surface, and *N* is the number of passes of the tool over the workpiece.

To be efficiently programmed on the computer, the deconvolution process is reduced to straightforward algebra. A wavelet-based algorithm has been developed and can be expressed as [269]:

(A2) $$R\left(x,y\right)=\sum_{n=0}^{\infty }\sum_{m=0}^{\infty }r_{nm}^{\left(\lambda_{Rx},\lambda_{Ry}\right)}\delta_{\lambda_{Rx}}{}^{n}\left(x\right)\delta_{\lambda y}{}^{m}\left(y\right)$$

where $\delta_{\lambda_{Rx}}{}^{n}\left(x\right)$ and $\delta_{\lambda_{Ry}}{}^{m}\left(y\right)$ are the derivative of the Gaussian along *x* for *λ = λ_R__x_* and along *y* for *λ = λ_R__y_*, respectively.

The Fourier transform method is easy to implement and computationally efficient. However, the deconvolution to calculate dwell time function using Fourier transform is numerically unstable and divergent [270], and sometimes fails to converge, resulting in the residual ridge cannot be completely removed [271].

(2) The iterative method uses the iterative approximation method in numerical computation. An initial function is assigned to the dwell function, then the residual error after convolution is calculated to obtain a new dwell function. Iterative loop calculation was carried out until the convergence condition is met. The Bayesian principle [272], Van Cittert method [273], Lucy-Richardson algorithm [274], Tikhonov regularization [275], self-adaptive algorithm [135], and resistance algorithm [276] were used to carry out the iterative method.

The numerical iterative method is easy to implement, but computationally efficiency is low. In some cases, it fails to converge because of concussion.

(3) The matrix method transforms the convolution into a matrix product to solve the dwell time [277] to expand the freedom of the solution [278]. The actual material removal amounts on a given point can be described as:

(A3) $$R_{a}\left(x_{k},y_{k}\right)=\sum_{i=1}^{N_{i}}A\left(x_{k}-\xi_{i},y_{k}-\eta_{i}\right)t_{c}\left(\xi_{i},\eta_{i}\right)$$

where *N_t_* is the total number of ion beam dwell points, *A*(*x_k_*-*ξ_i_*,*y_k_-η_i_*) is the material removal amount per unit time at point (*x_k_*,*y_k_*) when the center of the ion beam dwell is on the point (*ξ_i_,η_i_*), and *t_c_*(*ξ_i_,η_i_*) is the dwell time.

In matrix form, Equation (A3) can be expressed as:

(A4) $$\left(\begin{array}{c} r_{a_{1}}\\ r_{a_{2}}\\ \ldots \\ r_{a_{N_{r}}} \end{array}\right)=\left(\begin{array}{cccc} a_{11} & a_{12} & \ldots & a_{1N_{t}}\\ a_{21} & a_{22} & \ldots & a_{2N_{t}}\\ \ldots & \ldots & \ldots & \ldots \\ a_{N_{r}1} & a_{N_{r}2} & \ldots & a_{N_{r}N_{t}} \end{array}\right)\left(\begin{array}{c} t_{1}\\ t_{2}\\ \ldots \\ t_{N_{t}} \end{array}\right)$$

The minimal least-squares solution [277], nonnegative least-squares method [279], subspace Barzilai-Borwein gradient algorithm [280], Tikhonov regularization [281], and least square QR decomposition method [282] can improve the calculation results when using the matrix method.

The matrix method is based on the following assumptions [188]: (a) Material removal is linear and proportional to dwell time. (b) The removal function is constant over the entire surface and over time. By far the most common approach for calculating dwell time distribution is the matrix method.
